# Design, Implementation, and Advances in Indirect SERS Sensors for Biomedical and Human-Health-Related Analyte Detection

**DOI:** 10.3390/s26061999

**Published:** 2026-03-23

**Authors:** North Pinkley, Uchhwas Banik, Nayeem Anam, Aastha Oza, Kevin J. Ledford, Bhavya Sharma

**Affiliations:** Department of Chemistry, University of Tennessee Knoxville, 1420 Circle Drive, Knoxville, TN 37966-1600, USA; npinkley@vols.utk.edu (N.P.); ubanik@vols.utk.edu (U.B.); nanam@vols.utk.edu (N.A.); kledfor8@vols.utk.edu (K.J.L.)

**Keywords:** biosensing, nanotag, nano sensors, SERS, multiplexing, point of care, lateral flow device, immunoassay

## Abstract

**Highlights:**

**What are the main findings?**

- This review provides an overview of SERS nanotags, explaining their structural design, functional components, and underlying mechanisms for Raman signal enhancement.
- This work summarizes the applications of SERS nanotags for the detection and diagnosis of human-health-related analyte detection, highlighting recent trends in assay design and application.

**What are the implications of the main findings?**

- By unifying the fundamental principles and current biosensing applications of SERS nanotags, this review serves as a broad overview for researchers seeking to design, apply, or further develop SERS-based nanosensors.
- The insights presented help identify key contributors, emerging trends, and future directions in SERS nanotag research, promoting collaboration and accelerating translation of these technologies into clinical and real-world use.

**Abstract:**

Novel, accurate molecular diagnostics are driving new advances across medicine, public health, and environmental monitoring. Surface-enhanced Raman spectroscopy (SERS) nanotags are powerful platforms for ultrasensitive, multiplexed, and quantitative detection of molecular targets. This review focuses on indirect sensing strategies, where SERS nanotags act as signal transducers, resulting in enhanced and unique Raman spectra upon binding of target analytes (high specificity) and allowing for ultralow limits of detection. These indirect SERS sensors typically consist of a plasmonic core, a Raman reporter molecule, and a ligand that targets the analyte of interest. Each of these components contributes to the sensitivity, stability, and selectivity of the system. Rational design of SERS nanotags requires balancing enhancement efficiency with reproducibility, biocompatibility, and assay integration. The choice of reporter molecules, for instance, governs spectral uniqueness and enables multiplexed detection of multiple analytes within a single sample. Recent advances in artificial intelligence and machine learning are accelerating nanotag development by enabling predictive control over nanostructure geometry, composition, and optical response. SERS nanotags are increasingly being integrated into diagnostic formats, such as lateral flow assays and microfluidic devices, offering both qualitative and quantitative analysis at the point of care. This review provides an overview of key design principles, common strategies for nanostructure functionalization and stabilization, and emerging biosensing applications, serving as a practical guide for researchers seeking to design and implement SERS nanotags.

## 1. Introduction

Advances in medicine are being driven by the development of new molecular-based technologies for disease diagnostics, imaging, and therapeutics [1,2]. These interrelated fields are aiding in improving the understanding of the mechanisms of disease, which leads to early and enhanced detection, as well as the discovery of new therapeutic targets. Additionally, advances in these fields, combined with artificial intelligence and machine learning approaches, are revolutionizing healthcare and the development of precision medicine [3,4,5]. Molecular diagnostics involves the monitoring of biomarkers, including proteins, nucleic acids, small molecules, and metabolites. Conventionally, biomarkers are monitored through techniques such as PCR, ELISAs, HPLC, mass spectrometry, and other lab-based testing methods. Conventional tests often require multi-step sample processing, long analysis times, and non-portable equipment. Wearable sensors, i.e., smart watches, fitness devices, continuous glucose monitors, etc., have limited selectivity and specificity, and issues with reusability and handling of large datasets [6,7,8,9,10]. There are also limited wearable chemical sensors for monitoring neurological health [11]. There is an unmet need for sensors that are highly sensitive and specific, rapid, low-cost, quantitative, and that require little to no sample preparation.

Raman spectroscopy is an inelastic light scattering technique that measures the frequency shift between incident laser photons and those scattered by a sample. These frequency shifts correspond to molecular vibrations that are highly specific to individual chemical bonds and molecular structures, enabling precise molecular fingerprinting [12]. Compared with other analytical techniques, Raman spectroscopy offers several advantages: it enables rapid, specific, and non-destructive analysis; requires minimal sample preparation; and can be implemented using portable instrumentation for on-site or in-field measurements [13]. Despite these advantages, conventional Raman spectroscopy suffers from inherently low sensitivity because Raman scattering is an intrinsically weak process; only about one in every 10^8^ photons is Raman scattered. This limitation renders normal Raman spectroscopy unsuitable for detecting low-abundance analytes, such as trace biomarkers in complex biological matrices, where weak target signals are easily masked by fluorescence or background contributions [14]. To overcome this sensitivity barrier while retaining many of the inherent advantages of regular Raman, surface-enhanced Raman spectroscopy (SERS) has emerged as a powerful alternative.

**Figure 1:**
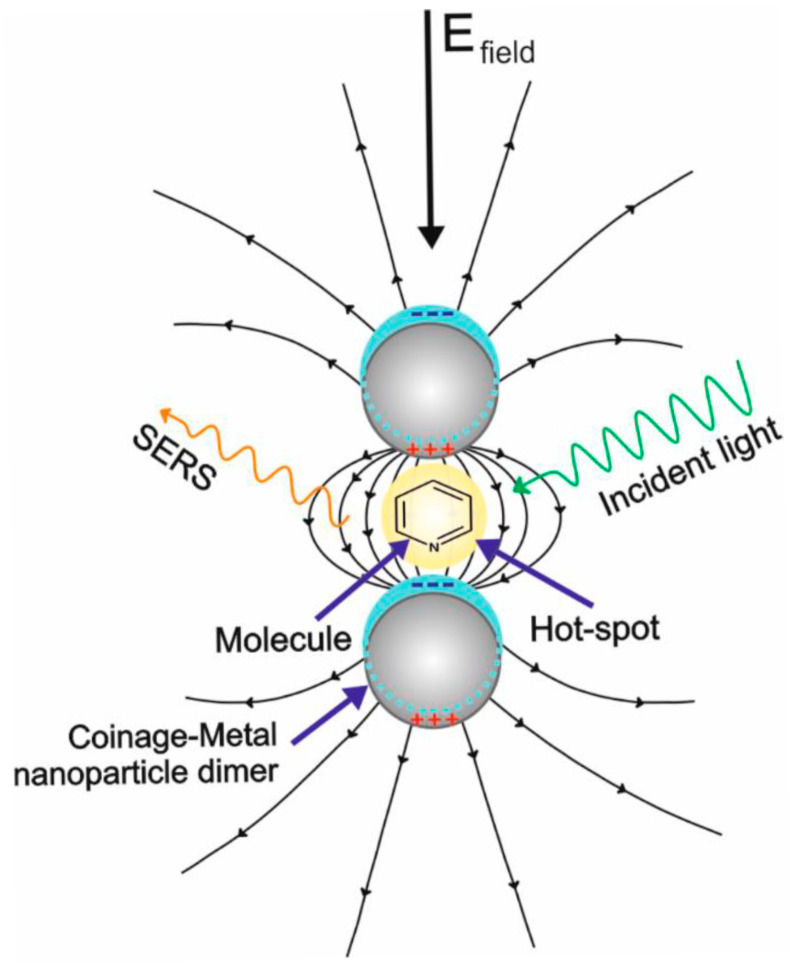
Schematic showing the hotspot effect generated by the localized plasmon resonance generated between two nanoparticles [17]. The arrows shows the direction of electric field, and the (+) and (−) symbols denotes the positive and negative surface charge, respectively. Reproduced from Ref. [17].

SERS enhances Raman signals by exploiting localized surface plasmon resonance (LSPR), a phenomenon that occurs when conduction electrons in a noble metal nanostructure collectively oscillate in response to incident light [15]. This oscillation generates intense, localized electromagnetic fields near the metal surface that can amplify the Raman scattering of molecules in close proximity by several orders of magnitude. When two or more plasmonic nanostructures are close enough for their electric fields to overlap, “hotspots” can form (Figure 1), which can generate signal enhancements of up to 10^10^-fold [16].

**Figure 2:**
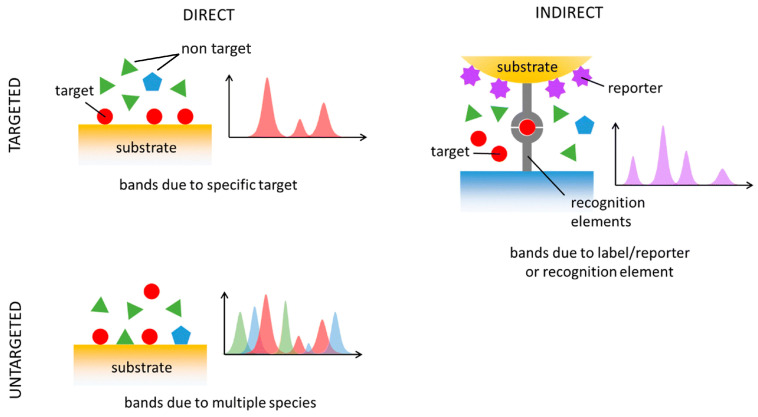
Comparison of direct (**left**) and indirect sensing (**right**) for targeted (**top**) and untargeted (**bottom**) modalities [18]. Reproduced from Ref. [18].

SERS-based sensing can be broadly classified into direct and indirect detection modes (Figure 2). In direct SERS, the analyte of interest is directly brought to the metal surface, producing an enhanced Raman spectrum that reflects the intrinsic molecular vibrations of the target analyte. However, many biologically relevant molecules have a low affinity for metal surfaces or exhibit overlapping vibrational signatures, complicating spectral interpretation in complex biological samples using direct sensing [14]. Consequently, indirect SERS approaches are commonly employed in biosensing applications, where signal transduction is achieved via a well-defined Raman reporter molecule rather than the analyte itself [18].

This review will focus on the design of SERS nanotags and the application of the nanotags for indirect SERS sensing. SERS nanotags are primarily nanoparticle (NP)-based sensors used to detect analytes with high specificity. SERS-based sensors can be applied for imaging [19], precision drug delivery [20], and photothermal therapy [21]; however, this review will focus on the applications of SERS sensors for diagnostic and analyte monitoring published within the past decade. SERS nanotags appear in the literature under different names, such as “SERS tags,” “bioconjugate probes,” and “nanosensors,” [22,23,24]. In this review, the term “nanotags” will be used for clarity and consistency. A SERS nanotag is typically comprised of three essential components: (1) a plasmonic nanostructure (such as a gold or silver nanoparticle) that provides the source of electromagnetic enhancement, (2) a Raman reporter molecule that generates a strong, distinct, and quantifiable vibrational signal, and (3) an analyte-specific targeting ligand (i.e., antibody, aptamer) that enables selective binding or recognition of the target analyte. The design of an effective SERS nanotag requires careful consideration of each component to optimize signal enhancement, stability, and analytical performance.

When an SERS nanotag binds to its target, nanoparticle aggregation or clustering can occur, creating additional electromagnetic hotspots that further amplify the Raman signal. The resulting increase in reporter signal intensity correlates directly with analyte concentration, enabling quantitative detection [25]. However, for alternative sensing methods such as competitive or displacement-based assays, higher analyte concentrations can lead to a decrease in reporter signal intensity as the analyte displaces reporter molecules or inhibits nanoparticle aggregation. In both cases, quantitative analysis is achieved by constructing calibration curves that relate analyte concentration to the integrated intensity of the reporter peak, allowing unknown sample concentrations to be determined from the fitted response [25]. This quantitative capability, arising from the precise interplay of nanostructure design, reporter selection, and surface chemistry, forms the foundation for reliable SERS-based sensing.

Through their high sensitivity, molecular specificity, and versatility in functionalization, SERS nanotag assays have emerged as powerful and adaptable sensing platforms for biological detection. Their ability to convert molecular recognition events into quantifiable optical signals has positioned SERS-based sensors as a promising tool for various applications, including point-of-care diagnostics, biomedical imaging, and drug monitoring [20,26,27]. This review will focus on the design, implementation, and advances of SERS sensors used for small molecule sensing and disease diagnostics.

## 2. Plasmonic Nanostructure

The first component of the SERS nanotag is the plasmonic nanostructure, which provides the electromagnetic enhancement necessary to generate a strong Raman signal from the reporter molecule. Since the inception of SERS in 1977, researchers have explored a wide variety of metals and morphologies to optimize plasmonic performance. Gold (Au) and silver (Ag) are the most frequently used metals for SERS nanostructures. Among these, gold and silver have become the dominant materials because their localized surface plasmon resonance (LSPR) bands can be tuned within the visible to near-infrared (NIR) range. These are the spectral regions where Raman scattering is least affected by fluorescence background and where biological samples are relatively transparent.

### 2.1. Spherical Nanoparticles

**Figure 3:**
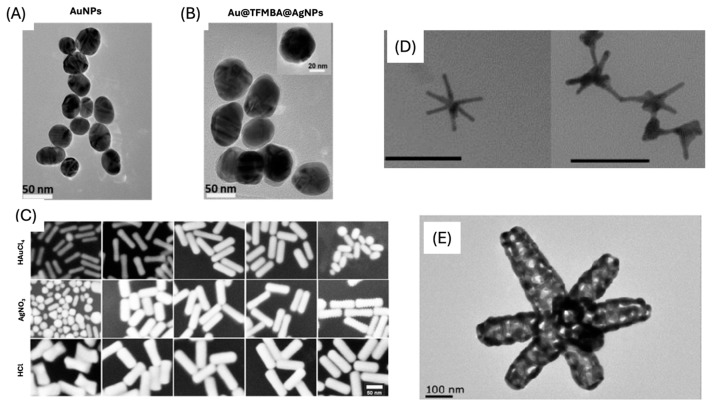
TEM images of (**A**) Au NPs and (**B**) Au@TFMBA@Ag NPs [35]. (**C**) SEM images of AuNRs [36]. (**D**) TEM images of the AuNS [32]. (**E**) TEM image of a typical Au nanocage [37]. (**A**,**B**) were adapted from Ref. [35]. (**C**) adapted with permission from Ref. [36]. (**D**) adapted from Ref. [32]. (**E**) adapted from Ref. [37].

Colloidal gold nanoparticles (AuNPs) are a widely used plasmonic substrate for SERS nanotags (Figure 3A). Their popularity is due to the simple and reproducible synthesis, the chemical stability/inertness of gold, and the well-established methods available to control particle size and surface chemistry [28]. Typically, spherical AuNPs are synthesized by reducing tetrachloroaurate with trisodium citrate dihydrate, a method that allows for fine control over particle size through the stoichiometry of the reducing agent, reaction times, and temperature. Because of these favorable characteristics, AuNPs have been applied extensively in a variety of biophotonic applications, including SERS biosensing [29]. While gold is the most employed material, silver nanoparticles (AgNPs) also offer exceptionally strong plasmonic activity and can produce higher electromagnetic enhancement factors due to their sharper plasmon resonances. AgNPs also have quick, reliable, and tunable syntheses, making them preferable SERS substrates [30]. However, AgNPs are generally less chemically stable than AuNPs, tending to oxidize under ambient or biological conditions, which can alter their optical properties and limit their long-term use.

### 2.2. Anisotropic Nanoparticles

The chemical, optical, and electrical characteristics of NPs can change depending on their form. Beyond simple spheres, researchers have designed nanostructures with diverse morphologies, such as nanostars, nanorods, nanocubes, and nanoprisms [31]. Non-spherical NPs are considered anisotropic nanostructures (Figure 3). The properties of anisotropic NPs vary depending on the shape of the NPs, in contrast to spherical NPs, which have homogeneous qualities in all directions [31]. Among these anisotropic structures, nanostars (NSs) are a widely used substrate due to the fact that they have been shown to possess higher SERS enhancement capabilities compared to spherical NPs [32]. This increased enhancement is due to the strong electromagnetic field around the tips of the star, indicating more intense “hot spot” areas. Structures featuring sharp edges, tips, or interparticle gaps can generate stronger localized electromagnetic fields and create highly efficient SERS hotspots [33]. Another common type of anisotropic structure used is gold nanorods (AuNRs). These structures are unique because they have dual-mode LSPR maxima, arising from the transverse (perpendicular to the long axis) and longitudinal (along the long axis) plasmon modes, which are tunable through changing the aspect ratio (ratio of length to diameter) of the nanorods [34].

### 2.3. Core-Shell Nanoparticles

Synthesis of core-shell nanoparticles is a common element in the development of SERS nanotags. Core-shell morphologies allow for RRMs bound to the core nanoparticle to be enhanced through the exploitation of the dual plasmonic effect, which arises when the LSPR from two metals overlap and hybridize to form an intense electric field between the two metal surfaces [38]. Additionally, embedding RRMs between the core and the shell can minimize desorption from the surface and help maintain the SERS signal for nanotags over extended periods of time [39]. Another common core-shell nanoparticle geometry is the combination of a metal shell with iron oxide core nanoparticles, which allows for the magnetic properties of iron oxide to be combined with the plasmonic properties of the metal shell [37]. Analytes of interest are bound to the surface of the magnetic nanoparticles, and then magnets are used to pull the analyte-bound nanotags from solution. Through this separation and isolation of the analytes, the limit of detection (LOD) can be lowered. To further lower LODs, nanoparticles are coated in silica, which protects adsorbed RRMs and prevents their dissociation from the metal surface, stabilizing the nanoparticles and resulting in increased SERS signal [25].

### 2.4. Supported Nanoparticles

Supported nanoparticles form a major class of SERS substrates in which plasmonic metals are immobilized on higher-dimensional materials to create stable, high-density hotspots for sensitive molecular detection. Graphene oxide (GO) is a 2D carbon structure to which AuNPs are easily attached (GO@AuNP) and are a common example of a type of supported morphology. GO provides a stable surface that reduces nanoparticle aggregation and increases SERS enhancement due to the electronic interaction between the surface plasmons and pi-conjugated domains of GO [40]. There are many other 2D and 3D structures conjugated with NPs that have been used to increase stability, optimize electrochemical enhancement, or facilitate analyte detection, such as metal-organic frameworks (MOFs), carbon-dots, polymers, paper, etc. [41].

Here, we summarize the major classes of plasmonic nanostructures commonly employed for SERS sensing. Rational control over nanoparticle shape, size, and interparticle spacing is essential for optimizing electromagnetic enhancement and overall sensing performance. Although precise experimental tuning of these parameters remains challenging, recent progress in computational modeling, high-throughput synthesis, and data-driven analysis has enabled more predictive design of plasmonic architectures with tailored optical properties. Machine-learning approaches trained on simulated and experimental datasets are now being used to uncover structure–property relationships, optimize synthetic conditions, and guide the fabrication of nanostructures with user-defined plasmonic responses [42]. Together, these advances offer a powerful route toward intelligently tunable, application-specific SERS substrates.

## 3. Raman Reporter Molecules

In SERS nanotags, signals from Raman reporter molecules (RRMs) are critical for the indirect quantification of target analytes. RRMs are chosen based on several key characteristics: strong and unique Raman peaks, a large Raman scattering cross-section, chemical stability, and compatibility with nanoparticle surface chemistry. When multiple nanotags bind to a target, the proximity of their plasmonic nanostructures generates SERS “hotspots,” dramatically enhancing the Raman signal of the attached reporter molecules. The intensity of the RRM correlates directly with the amount of target present, enabling quantitative analysis through calibration curves that relate RRM intensity or peak area to analyte concentration [25]. Selecting an appropriate RRM is therefore essential, as it determines signal strength, spectral reproducibility, and assay sensitivity. RRMs used in nanotags generally fall into two major categories: dye-based and non-dye-based molecules. A full table of common RRMs along with their structures is provided in Table 1.

Optimal SERS enhancement occurs when RRMs form a uniform monolayer or sub-monolayer on the nanoparticle surface. This means that during nanotag fabrication, parameters influencing surface monolayer formation are particularly critical. These include reporter concentration, incubation time, solvent composition, pH, ionic strength, and the presence of competing ligands or stabilizers (e.g., citrate, CTAB, PVP), all of which affect surface coverage, molecular orientation, and packing density [43,44,45]. Reporter molecules containing thiol (–SH) and amine (–NH_2_) groups are often favored due to their strong binding affinity for gold and silver nanoparticles, enabling stable attachment and controlled monolayer formation [43]. Incomplete ligand exchange, multilayer formation, and desorption under physiological conditions can all introduce spectral variability and batch-to-batch inconsistencies in SERS sensors [46,47]. It is essential to optimize co-absorption strategies with the selected targeting ligand to ensure optimal signal intensity is balanced with colloidal stability and ligand accessibility [19].

### 3.1. Dye RRMs

Dye molecules and aromatic chromophores are commonly used as RRMs because they possess extended conjugated π-electron systems, which produce strong Raman scattering due to increased molecular polarizability. These dyes exhibit intense, well-defined vibrational modes and often are in resonance with the excitation wavelength, further enhancing the SERS signals. Dye RRMs are valued for their high sensitivity, distinct spectral features, and robust signal output, making them especially suitable for indirect detection formats in which the RRM serves as the quantifiable element of the assay. Common dye RRMs include Cyanine 5 (Cy5), malachite green (MG), Rhodamine 6G (R6G), and Nile Blue A (NBA). Dye RRMs can operate bifunctionally, as both reporter molecules and targeting ligands. For example, Li et al. employed a modified Rhodamine B structure to bind and detect Hg^2+^ ions in contaminated milk samples [48].

### 3.2. Non-Dye RRMs

**Figure 4:**
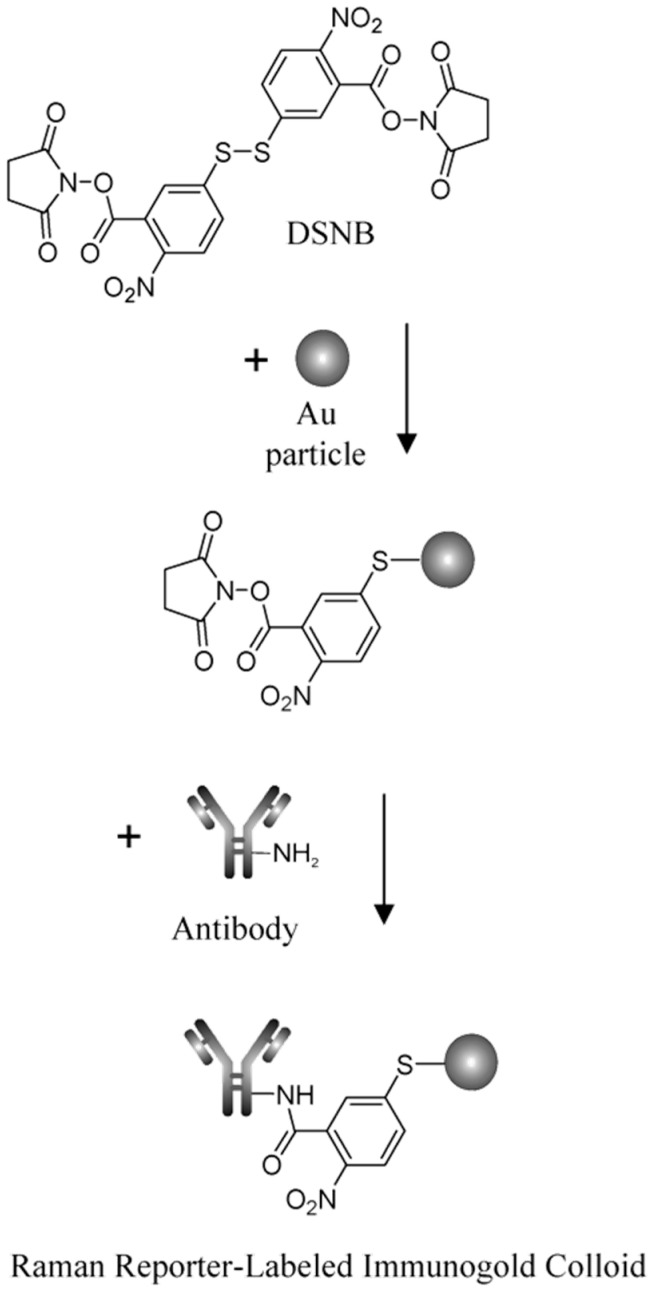
Scheme of how DSNB functions as a reporter and a linker molecule to covalently attach antibodies to gold nanoparticles. The arrows indicate the reaction pathway to covalently link an antibody to a gold nanoparticle using DSNB. [49]. Reproduced with permission from Ref. [49].

Non-dye RRMs derive their Raman activity from specific chemical moieties, such as thiols, amines, nitro groups, nitriles, and aromatic rings. These structures generate strong, characteristic Raman peaks. These RRMs provide highly reproducible spectral signatures and can be chemically tailored to serve additional roles. In many designs, non-dye reporters act as bifunctional linkers: one functional group binds the nanoparticle surface, while another reacts with targeting ligands such as antibodies, peptides, or aptamers. This dual functionality simplifies fabrication and ensures a well-defined geometry between the plasmonic core, the reporter, and the biorecognition element. For example, the Porter group at the University of Utah has popularized the use of the DSNB molecule (Figure 4) as a bifunctional linker where the thiol group binds to the metal surface, the nitro group provides a strong and distinct Raman signal, and an antibody is covalently attached using the succinimide group [49].

### 3.3. Multiplexing RRMs

**Figure 5:**
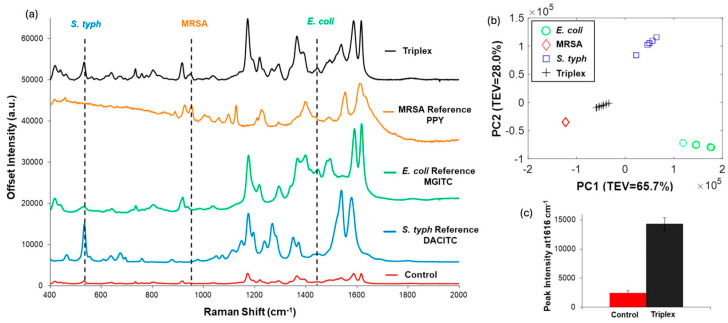
(**a**) Multiplex SERS using three unique RRMs (PPY, MGITC, DACITC) to distinguish three species of bacteria (MRSA, *E. coli*, and *S. typh*) visually, (**b**) Principal component analysis for the three species, and (**c**) difference of AOC of the characteristic peak between control and triplex [24]. Reproduced with permission from Ref. [24].

Multiplex SERS employs two or more RRMs with non-overlapping vibrational signatures to enable the simultaneous detection of multiple analytes. Each RRM is incorporated into a distinct nanotag population, and their combined spectra allow for parallel identification/quantification from a single measurement. Effective multiplexing requires RRMs that provide strong, narrow, and well-separated peaks that remain distinguishable even in complex samples. Sometimes chemometric approaches such as PCA combined with partial least squares regression (PLS-R) can be used to separate overlapping features and enhance the reliability of multiplex detection. Kearns et al. developed a multiplex approach with three unique RRMs on SERS tags to detect three different pathogenic bacteria with PCA (Figure 5) [24]. Other authors also used this multiplexed RRMs technique to detect multiple analytes for cancer [50,51,52,53,54,55], cardiovascular diseases [56,57,58,59], and traumatic brain injury [60,61]. Multiplexed RRMs significantly expand the capabilities of SERS nanotags, offering higher information density, improved diagnostic throughput, and the ability to analyze multiple biomarkers or chemical targets simultaneously.

## 4. Targeting Ligand

Choosing an effective target ligand while designing an SERS nanotag is another important step in the nanotag synthesis process. The type of targeting ligand (antibody, aptamer, or other) is key in determining the specificity and sensitivity of the nanotag. Other considerations include selecting the proper conjugation method, stabilization method, and assay design to match the desired targeting ligand. All these considerations must be combined and optimized to create a nanotag that is specific and effective for detecting the target analyte.

### 4.1. Antibody

Antibodies are widely used ligands for SERS nanotags. Antibodies are y-shaped proteins that have high affinity and specificity for antigens. Antibodies are produced by many organisms as part of their adaptive immune system against pathogens, such as viruses or bacteria. However, advances in microbiology and molecular biology have allowed scientists to generate antibodies against almost any target of interest [62]. Antibodies have distinct binding pockets that utilize non-covalent interactions to form a very specific interaction with an antigen. Due to this specificity, multiple antibodies can bind to the same antigen at different sites, allowing for an antigen to be “sandwiched” between two antibodies. For SERS nanotags, IgG-type antibodies are typically used; however, some groups have explored using antibody variants, such as single-chain variable fragments (scFv) or nanobodies, as targeting ligands [63,64].

### 4.2. Aptamer

Aptamers are short, single-stranded DNA or RNA sequences that fold into unique three-dimensional structures capable of binding molecular targets with high affinity and specificity. Unlike antibodies, which possess fixed tertiary structures, aptamers adopt multiple conformations depending on their local environment, and this dynamic folding enables selective recognition through a combination of hydrogen bonding, electrostatic interactions, and van der Waals forces [65]. Aptamers are relatively easy to generate using the Systematic Evolution of Ligands by Exponential Enrichment (SELEX) process, an iterative in vitro selection method in which large randomized nucleic-acid libraries are enriched toward sequences that bind a desired target. Compared with antibodies, aptamers offer several advantages, including low-cost chemical synthesis, excellent batch-to-batch reproducibility, long-term stability, and the ability to regenerate their binding activity after denaturation [65]. For SERS nanotag construction, aptamers are commonly conjugated to plasmonic nanostructures through the incorporation of thiol-modified oligonucleotides into the aptamer sequence for conjugation via gold-sulfur bonding. Other conjugation approaches are very similar to antibody conjugation strategies, including covalent binding through EDC (1-ethyl-3-(-3-dimethylaminopropyl) carbodiimide hydrochloride) and NHS (N-hydroxysuccinimide) coupling chemistry. Despite their benefits, aptamers can be limited by susceptibility to nuclease degradation and by the sensitivity of their binding performance to environmental factors that alter their tertiary structure. Aptamer design and generation have been enhanced by advances in computational approaches. For example, Quarin et al. utilized an automated computational program to systemically improve their aptamer-based catalytic sensor for SERS-based detection of SARS-CoV-2 and malaria genetic biomarkers [66].

### 4.3. Other Targeting Ligands

While antibody and aptamer-based nanotags are extremely common and most prevalent for the scope of this work, there are other types of targeting ligands that can be used to bind and indirectly detect target analytes. One emerging targeting ligand is the molecularly imprinted polymer (MIP), which is a highly stable synthetic polymer engineered with a specific binding pocket to mimic an antibody [67]. Another major class includes non-antibody proteins like enzymes, receptor proteins, and binding proteins that can bind selectively to cell receptors, metal ions, and other biologically relevant targets. Finally, host–guest ligands are supramolecular molecules (e.g., cyclodextrins, cucurbiturils, crown ethers) that selectively bind target analytes through size- and shape-complementary noncovalent inclusion interactions, enabling stable and reversible recognition for indirect SERS detection. These alternative targeting ligands have been mentioned here to provide a broad overview of possible ligands; however, they are not commonly applied to the indirect sensing applications detailed in this work.

### 4.4. Conjugation Strategies

The conjugation of targeting ligands to plasmonic nanostructures is a fundamental step in SERS nanotag fabrication, as it determines biological specificity, as well as influencing colloidal stability and signal reliability. The selected ligand must preserve both the optical enhancement properties of the plasmonic core and the functional integrity of the Raman reporter molecules. Inadequate or poorly controlled ligand conjugation can result in reduced binding efficiency, substrate aggregation, or attenuation of electromagnetic enhancement due to excessive interfacial spacing. Accordingly, multiple strategies have been developed to balance stability, control over ligand orientation, and reproducibility. The following sections summarize and compare the primary conjugation approaches used in SERS nanotag synthesis for both antibodies and aptamer-type ligands.

#### 4.4.1. Direct Adsorption

**Figure 6:**
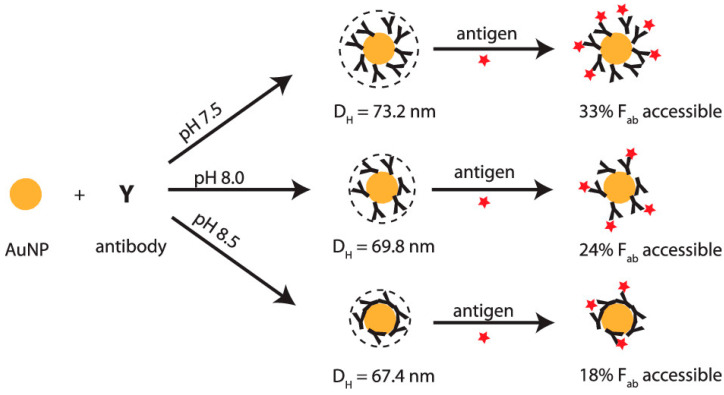
Figure showing how pH impacts antibody orientation during direct conjugation with AuNPs [69]. Reproduced with permission from Ref. [69].

The simplest way to conjugate a plasmonic substrate with antibodies is through direct adsorption via electrostatic interactions [68]. In direct adsorption, the positively charged region of the antibodies interacts with the negatively charged surface of a nanoparticle, which arises from the reducing agent used during nanoparticle synthesis (such as trisodium citrate) [28]. Additionally, thiol bonds between the cysteine groups within antibodies and the Au or Ag nanoparticle surface can react, thereby impacting adsorption. To aid in the absorption of antibodies to the nanoparticle surface, pH can be adjusted (Figure 6) to maintain the negative charge of the nanoparticle surface and facilitate electrostatic interactions [69].

#### 4.4.2. Linker Aided Direct Adsorption

**Figure 7:**
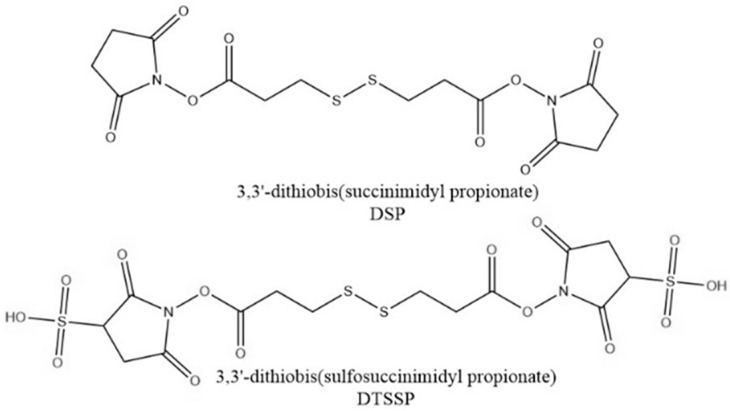
Structure of DSP and DTSSP. Structures generated in ChemDraw (ChemDraw Professional 22.2).

Another method for conjugation of antibodies to the surface of nanostructures is through a linker molecule. Linker molecules can be homobifunctional or heterobifunctional. Homobifunctional linkers have the same reactive moiety on each side of the molecule, while heterobifunctional linkers have different reactive moieties on each side of the molecule. DSP [3,3′-dithiobis(succinimidylproprionate)] (Figure 7), sometimes also denoted as DTSP, has been used as a homobifunctional linker to conjugate antibodies to reporter-labeled nanoparticles. The NHS ester moieties react with amine groups of lysines within antibodies to form stable amide bonds. The thiol groups adsorb to the surface of nanoparticles via Au/Ag-S bonds. Bashir et al. adsorbed DSP to mesoporous AuNPs to conjugate anti-PLAP for ovarian cancer [70]. DSP has similarly been used to conjugate different antibodies to AuNPs [71] and Au@AgNPs [35]. DTSSP [3,3′-dithiobis(sulfosuccinimidylproprionate)] (Figure 7) is a water-soluble homobifunctional linker alternative to DSP. The sulfo-succinimidyl esters react with amines in the lysine groups in antibodies to create a stable amide bond. Antibodies are first incubated with DTSSP to form the amide bond, and then the di-thiol groups adsorb to the nanoparticle surface. Wang et al. activated multiple antibodies with DTSSP, which were then adsorbed to AuNPs [50]. Other groups have similarly utilized DTSSP with antibodies to conjugate reporter labeled AuNPs [72].

#### 4.4.3. Covalent Conjugation Strategy

**Figure 8:**
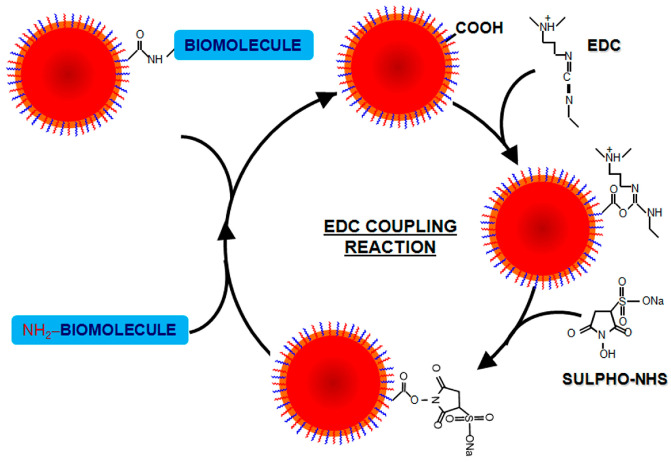
Scheme outlining EDC/NHS coupling reaction between nanoparticles and antibodies [76]. Adapted from Ref. [76].

Linker molecules can be modified via an EDC/NHS crosslinking coupling reaction; the mechanism is shown in Figure 8. EDC [1-ethyl-3-(-3-dimethylaminopropyl) carbodiimide hydrochloride] reacts with carboxylic acid moieties to form an unstable o-acylisourea intermediate. NHS (N-hydroxysuccinimide) and its water-soluble alternative sulfo-NHS react with the unstable intermediate to form an amine-reactive NHS (or sulfo-NHS) ester that readily reacts with amines to form stable amide bonds. When preparing EDC and sulfo-NHS solutions, allow the powders to equilibrate to room temperature; they also hydrolize rapidly in water, so solutions should be used within five minutes of preparation. The linker molecule must contain a carboxylic acid moiety and a moiety that adsorbs to gold/silver, such as sulfhydryl groups. The most common linker used to conjugate antibodies to nanostructures is HS-PEG-COOH [73,74,75].

Wang et al. adsorbed 11-mercaptoundecanoic acid (MUA) on AuNPs, activated the carboxyl groups with EDC/sulfo-NHS, and covalently coupled detection aptamers for different exosomes [52]. Others similarly used MUA or mixed MUA/PEG-COOH layers on AuNPs, followed by EDC/NHS activation to attach multiple antibodies to reporter encoded tags [51,77]. Additionally, silica-encapsulated Au nanostars were functionalized with 3-(triethoxysilyl) propyl succinic anhydride (TEPSA) to introduce surface carboxyl groups, which were then activated with EDC/sulfo-NHS to immobilize two aptamers [78]. Other strategies included coating Au@Ag-Au nanogap particles with a PEG-COOH thiol, activating the termini with EDC/NHS, and conjugating detection antibodies for digital SERS quantification [79] or first modifying carboxylated magnetic beads or Ag-Au nanoflowers with EDC/NHS before grafting capture and detection antibodies [54,80]. In core-shell systems, an ultrathin silica shell was grown on Au@Ag nanoparticles, aminated with APTES buffer, carboxylated with succinic anhydride, and finally incorporated EDC/sulfo-NHS chemistry to bind six different antibodies on a seven-channel ovarian cancer platform [81].

Covalent conjugation of bio-recognition elements of SERS nanotags has largely replaced non-covalent physisorption in the diagnostics platforms owing to its superior stability, orientation control and reproducibility. Although simple adsorption is still used for rapid proof-of-concept studies [82,83], it is prone to ligand desorption in serum, random antibody orientation, and high batch-to-batch variability. In contrast, covalent strategies based on thiol-Au bonds [68,74,84], EDC/NHS amide coupling [50,51,54,77,78,79,80] or DSP/DTSSP crosslinkers [50,51,81] provide robust linkage that retains >90–95% activity after weeks of storage and exposure to undiluted biofluids [53,68,81], while heterobifunctional spacers enable controlled Fc-region orientation that routinely improves functional affinity two-to four-fold. These features yield inter-batch variation typically below 8–10%, a prerequisite for the quantitative clinical cohorts [35,70,72,81,84,85]. Additional polydopamine or silica encapsulation after covalent attachment further enhances antifouling properties [68,73]. Consequently, covalent conjugation has become the standard for transforming SERS nanotags into reliable, clinically translatable probes for biomarker detection.

### 4.5. Stabilization

Uncoated nanotags have multiple limitations, including aggregation, toxicity, oxidation, instability, and non-specific binding of biomolecules to the surface. Any of these can impact the in vitro or in vivo applications. Protective surface coatings, such as silica, polymers, and biomolecules, have been utilized to minimize the limitations created using plasmonic nanostructures. In this review, we focus on two stabilizing agents: bovine serum albumin (BSA) and polyethylene glycol (PEG). These are the two most widely adopted and representative stabilization strategies in SERS nanotag design. BSA and PEG offer distinct advantages depending on the intended application of the SERS nanotag.

#### 4.5.1. BSA

Bovine serum albumin (BSA) is one of the most highly used molecules to prevent non-specific binding and aggregation of nanoparticles, while also increasing biocompatibility for SERS nanotags. BSA is a plasma protein, similar in structure to human serum albumin, that maintains extracellular fluid volume in the human body.

**Figure 9:**
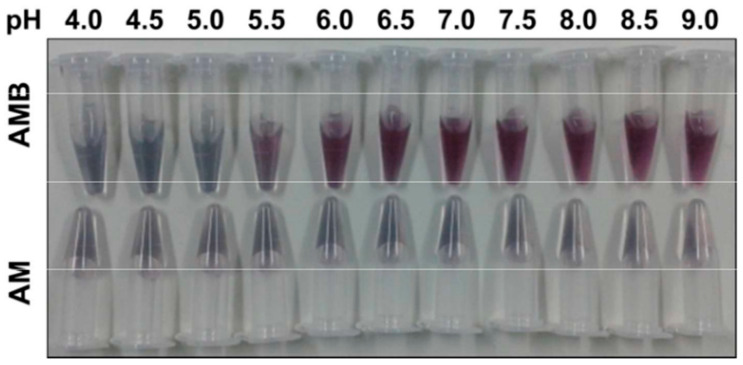
Difference between AuNP-RRM with (AMB) and without BSA coating (AM) in PBS over the pH range 4–9 [89]. Reprinted with permission from Ref. [89].

BSA serves as a universal stabilizing and blocking agent in virtually all SERS nanotag syntheses for diagnostic purposes. Added at 1–10% after reporter labeling and antibody/aptamer conjugation, BSA rapidly adsorbs onto any remaining bare Au/AgNPs surfaces, forming a protective protein corona that prevents aggregation in high-ionic-strength media and undiluted blood-based samples through combined electrostatic and steric repulsion. This simple step, explicitly used by many researchers, dramatically reduces non-specific binding (>90% background suppression), maintains colloidal stability for weeks to months, and ensures reproducible SERS performance in clinical samples, making BSA passivation an essential final protocol for translating nanotags into robust blood-based assays [51,61,81,86,87,88]. The volume and concentration are optimized based on the nanotag application. Zheng et al. compared AuNPs-RRM with and without a BSA surface coating over a range of pH values [89]. In Figure 9, AuNPs-RRM with BSA coating from pH 4–9 showed the red-wine color that is indicative of AuNP stability.

#### 4.5.2. PEG

Another common stabilizing agent is polyethylene glycol (PEG). PEG is a polymer that consists of a large chain of repeating ether subunits. Most commonly, PEG groups with thiol groups attached are used to promote ease of conjugation. Similarly, PEG derivatives with amine or carboxyl groups increase covalent surface modification in nanoparticles. The hydrophilic polyether backbone creates electrostatic protection, which prevents protein adsorption that is common in uncoated nanoparticles in physiological environments [90]. Additionally, the ether oxygen atoms form hydrogen bonds with surrounding water molecules, creating a hydration shell [91]. This shell helps prevent nanoparticles from interacting with other biological components, ultimately decreasing protein adsorption on the surface of the nanoparticle. This protection helps prevent aggregation of the nanotags, as well as increasing specificity towards the target molecule by limiting off-target binding. Thus, PEG is a vital stabilizing agent that provides many benefits in vitro and in vivo diagnostic applications.

Between these two stabilizing agents, BSA is widely favored for in-vitro diagnostic platforms due to its simplicity, low cost, and rapid adsorption-based blocking, which effectively suppresses aggregation and non-specific binding under physiological buffer conditions. In contrast, PEG provides covalently anchored, chemically defined surface modification with superior antifouling properties and long-term colloidal stability, making it more suitable for highly complex matrices and potential in vivo applications.

## 5. Integrating Nanotags into Assays

After nanotags have been carefully designed and synthesized, the final task is to implement them into a suitable assay format. Nanotags have been implemented in a wide variety of creative and useful assay formats, depending on the application and sample type. Implementing the correct assay format is key to reducing background signal and improving the interaction between SERS nanotags and the target analyte, with the goal of improving the limit of detection (LOD) of the nanotags. LOD is the standard measure of performance for a biosensor, referring to the lowest concentration of an analyte that can be distinguished from the blank (Equation (1)):

LOD = 3 × σ/m(1)

where sigma (σ) is the standard deviation of blank, and m is the slope of the calibration curve. Here, we will focus on summarizing the most common assay formats for detecting biomarkers and diseases using SERS nanotags.

### 5.1. Solution-Based Assay

The simplest format of the SERS nanotag assay is the solution-based assay. In a typical workflow for this type of assay, SERS nanotags are combined directly with the liquid sample of interest to detect the target analyte. The nanotags bind to the analyte in solution, forming aggregates or complexes that create strong electromagnetic “hotspots”, or the SERS signal is turned “on” or “off” after the targeting ligand binds to the analyte [18]. However, because biological and environmental samples often contain interfering substances that can dampen or obscure the SERS signal, many solution-based assays incorporate separation or washing steps to remove excess reagents and matrix components. Magnetic nanoparticles are particularly useful in this context, as they allow rapid and efficient magnetic separation of the target-bound complexes from the bulk solution, improving reproducibility and signal.

### 5.2. Immunoassays

Immunoassays, like lateral flow assays (LFA), have been implemented since the 1970s. However, the Porter group first integrated SERS and immunoassays together in 1999 to create an extremely versatile and powerful assay [92]. Today, immunoassays are a very common assay format used to implement SERS nanotags. Immunoassays primarily rely on antibody–antigen interactions; however, aptamers can be implemented as part of the SERS nanotag as the recognition element. There are multiple types and variations in immunoassays; here, we will focus on understanding the mechanism of the SERS-lateral flow assay (SERS-LFA) since this is the most common assay format for this type of sensing.

**Figure 10:**
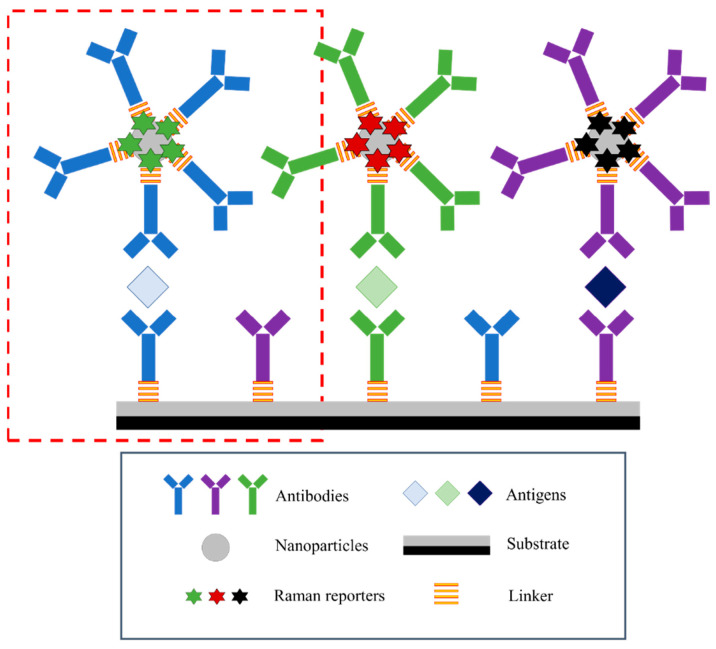
Scheme depicting the basics of an SERS sandwich assay. The frequently used approach enabling single-antigen analysis is shown in the red box [94]. Reproduced from Ref. [94].

**Figure 11:**
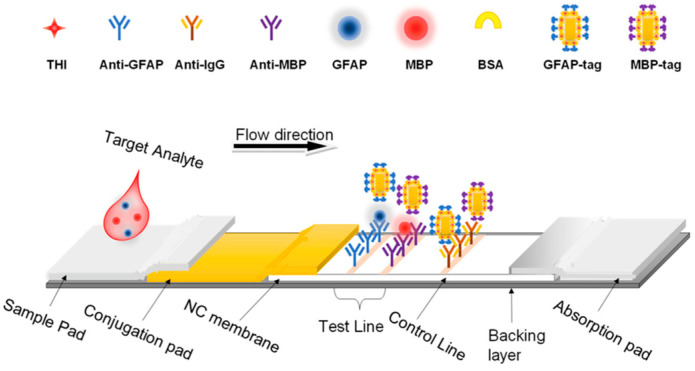
Schematic illustration of how an SERS-based LFA strip works for detecting brain injury biomarkers [36]. Reproduced with permission from Ref. [36].

SERS-LFA works in a very similar manner to an Enzyme-Linked Immunosorbent Assay (ELISA). First, a sample flows across a nitrocellulose test strip, and if present, the target analyte will bind with a capture antibody on the strip and subsequently a SERS nanotag to create a “sandwich” complex (Figure 10). The capture antibodies are deposited in a line across the test strip, and thus, when the target analyte is present, there will be an identifiable test line that can be scanned with a laser to determine the SERS signal intensity. Advantages of this assay include a rapid colorimetric result to determine if the target is present, combined with quantification of the analyte concentration through analysis of the SERS signal intensity. The SERS-LFA design (Figure 11) has been implemented for a variety of applications, including cancer and brain injury diagnostics. Current advances include using portable Raman spectrometers in conjunction with SERS-LFAs for point-of-care applications in clinical settings [93].

### 5.3. Microfluidic Assays

Microfluidic devices are powerful assay platforms for surface-enhanced Raman scattering (SERS)-based diagnostics. They offer precise fluid control and integration of sample preparation, mixing, and detection within a compact “lab-on-a-chip” system. By confining analytes in microscale channels, these devices enhance molecular interactions with SERS-nanotags, improving detection sensitivity and reproducibility [95]. Microfluidic systems can also be integrated with other assay platforms, including immunoassays and magnetic nanoparticle-based assays, to enhance nanotag interactions with the target and analyte separation, respectively. Together, these features make microfluidic SERS devices highly efficient, rapid, and versatile tools for sensitive and label-free detection in biomedical diagnostics.

## 6. Applications of SERS Nanotags

Having now discussed the composition of nanotags and various assay platforms, we now summarize recent and notable applications of SERS nanotags for the detection of biomarkers and diseases. We discuss applications of SERS nanotags to detect bacteria, viruses, cancer, cardiovascular diseases, traumatic brain injury, and food contaminants. We also provide a table summarizing the nanotag components, assay type, and LOD for each listed application.

### 6.1. Bacteria

**Figure 12:**
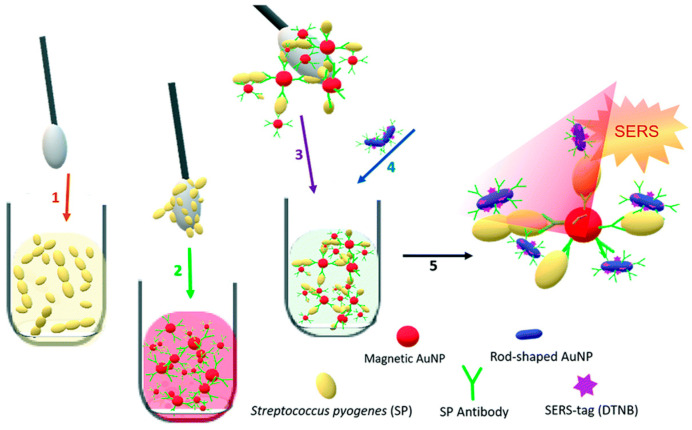
A swab-based approach to detect *S. pyogenes* (adapted with permission from Ref. [98]).

Rapid detection and identification of bacteria in food and patient samples are integral to human health and safety. SERS-based nanotags offer a compelling alternative to conventional culture-based or genetic tests for bacterial detection and identification, which are relatively slow and require specialized personnel [96]. Many recent SERS-based bacterial assays focus on rapid detection in clinical environments (Figure 12), including an aptamer-based SERS sensor for the detection of *E. coli* in urine samples for diagnosis of UTIs, where a 95% accuracy was achieved [97]. Other novel assays include SERS-LFAs for: rapid diagnosis of Group A *Streptococcus pyogenes* (GAS) from a throat swab [98], monitoring the concentration of *P. gingivalis* as a biomarker of oral health from saliva using a handheld Raman spectrometer for point-of-care applications [99], multiplexed identification and quantification of three different bacterial pathogens that are common sources of food-borne illness [100], and quantification of *E. coli* O157:H7 concentration from spiked Romaine lettuce [99]. These studies (summarized in Table 2) highlight the applications of SERS nanotags for rapid and multiplexed bacterial detection, for both human disease diagnosis and food contamination.

### 6.2. Virus

**Figure 13:**
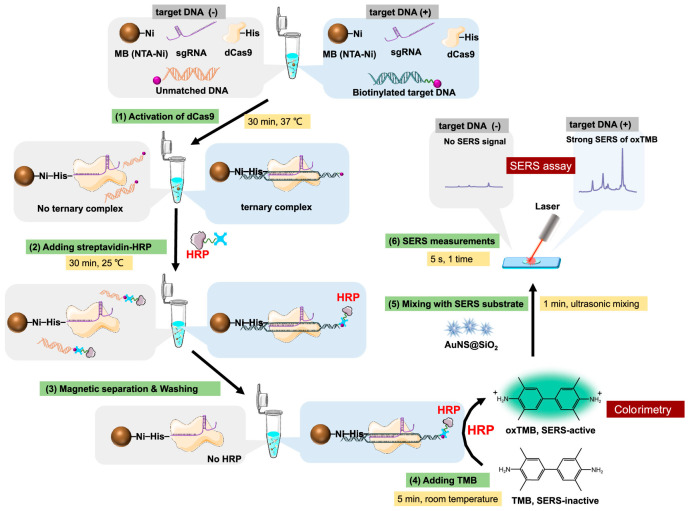
A CRISPR-based sensor that is used to amplify and detect hepatitis C RNA at low levels [107]. Reproduced from Ref. [107].

Detection of viruses is challenging due to their small size (~20–400 nm) and environmental resilience; SERS-based methods, however, offer a powerful approach to detect low viral loads. Reports on SERS viral detection have increased rapidly since the COVID-19 pandemic, with SERS sensing proposed as an alternative method for detection of the SARS-CoV-2 virus [17], including development of a magnetic microdroplet-based SERS assay to detect SARS-CoV-2 nucleocapsid protein at levels as low as 0.22 PFU/mL [105]. In addition to the SARS-CoV-2 virus, SERS-LFAs incorporating Ag@Au nanostars were used to quantify levels of anti-Influenza A antibody from both the colorimetric and SERS response of the assay [106], and a unique CRISPR-cas9-based assay (Figure 13) was developed to detect DNA from human papillomavirus (HPV) utilizing silica-coated nanostars as the plasmonic substrate [107]. SERS nanotags have also been employed for the detection of prevalent endemic tropical diseases, such as Zika, Dengue, and Monkeypox viruses, which are often difficult to diagnose in resource-limited tropical regions due to their overlapping symptoms, the need for rapid detection, and limited access to advanced diagnostic tools [63,108,109]. Viral detection using SERS nanotags has predominantly been applied to human viruses; however, there are now applications in development for plant and animal viruses. Kissell et al. implemented a hydrogel assay system in combination with SERS nanotags that rapidly uptakes viral RNA and detects the presence of tobacco mosaic virus (TMV) [110]. These distinct SERS-based viral assays highlight the versatility of SERS-based nanotags in accurately detecting and quantifying viral species across a variety of sample types, demonstrating their potential for widespread diagnostic applications (Table 3).

### 6.3. Cancer

**Figure 14:**
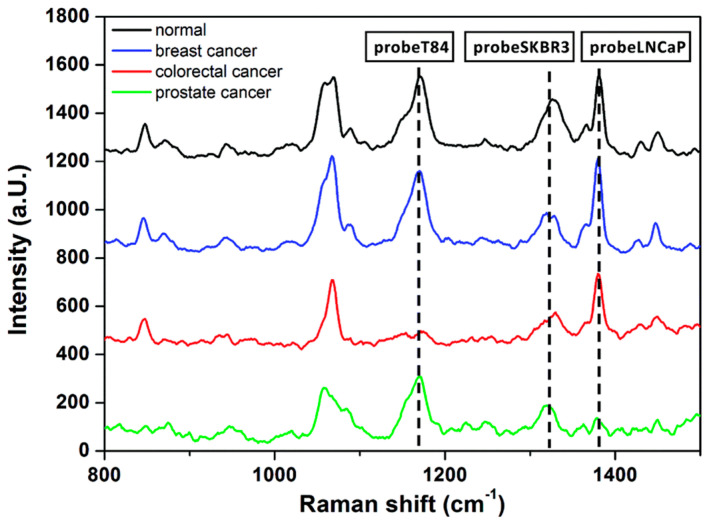
SERS spectra of the supernatant after different blood samples of cancer patients were added to the mixture of the capturing substrates and the SERS probes [52]. (adapted from Ref. [52]).

**Figure 15:**
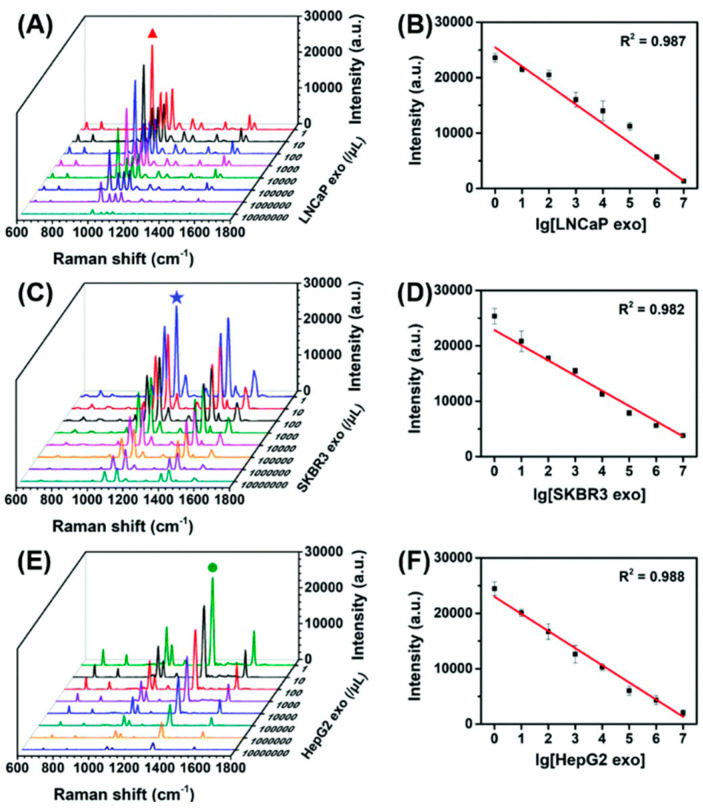
SERS spectra obtained for the SERS sensors with (**A**) LNCaP exosomes (1002 cm^−1^ characteristic peak for 2-Mpy), (**C**) SKBR3 exosomes (1140 cm^−1^ characteristic peak for 4-ATP), and (**E**) HepG2 exosomes (1335 cm^−1^ characteristic peak for NTP) with varying concentrations. Standard curve of the corresponding peak intensities I_1002_, I_1140_, and I_1335_ as a function of exosome concentrations for (**B**) LNCaP, (**D**) SKBR3 and (**F**) HepG2, respectively [53]. (adapted from Ref. [53]).

SERS nanotag assays have emerged as a versatile platform for non-invasive cancer diagnosis via liquid biopsy, targeting diverse malignancies, such as breast cancer [55,72,87] melanoma [50], lung cancer [35,84], ovarian cancer [70], prostate cancer [80,113,114], pancreatic cancer [68] osteosarcoma [51], gastric cancer [115], hepatocellular carcinoma [53], and multiple myeloma [71], while enabling accurate staging and monitoring of therapeutic response. Various research groups have developed innovative nanotag designs to achieve ultrasensitive multiplex detection of exosomal proteins, tumor antigens, and nucleic acids directly in clinical biofluids. The innovative assays include a signal-off magnetic assay using DTNB/2NAT/MMC-encoded AuNP tags targeting HER2, CEA, and PSMA exosomes with LODs ranging from 32 to 203 exosomes µL^−1^ [52] (Figure 14). Other examples are embedded-reporter gold–silver–silver heterostructure nanotags for displacement-based profiling of PSMA, HER2, and AFP exosomes with LODs of 26–72 exosomes µL^−1^ in serum [53] (Figure 15); polydopamine-wrapped PEARL tags for pancreatic markers, including MIF, GPC1, and EGFR, with an ultralow LOD of approximately one exosome in 2 µL serum [68]; MBA/DTNB/TFMBA-encoded AuNP tags on CD63-magnetic beads targeting EpCAM, GPC1, and CD44v6 pancreatic exosomes [54];.an electro-osmotic microarray with quadruple-reporter (MBA/TFMBA/DTNB/MPY) barcodes for tetraspanin and tumor-specific markers (MCSP/MCAM/ErbB3) for melanoma, lung, and breast cancer EVs (LOD 10^3^ EVs mL^−1^) [50].; MGITC-loaded Au@Ag core-shell tags in a seven-channel microfluidic system targeting CD81, CD9, EpCAM, EGFR, CD24, and CA125 on ovarian cancer EVs (LOD 10 particles mL^−1^ and AUC >0.94 for early-stage detection) [81]; and mesoporous Au nanotags combined with MOF-derived magnetic nanorods for MUC16, CLDN3, and FOLR1 in ovarian cancer (LOD 2.13 EVs µL^−1^) and demonstrated performance superior to CA-125 [85]. These diverse advancements in plasmonic nanostructures and encoding strategies highlight the robust potential of SERS nanotags for precise multiplexed molecular profiling in early cancer detection (Table 4).

### 6.4. Cardiovascular Diseases

**Figure 16:**
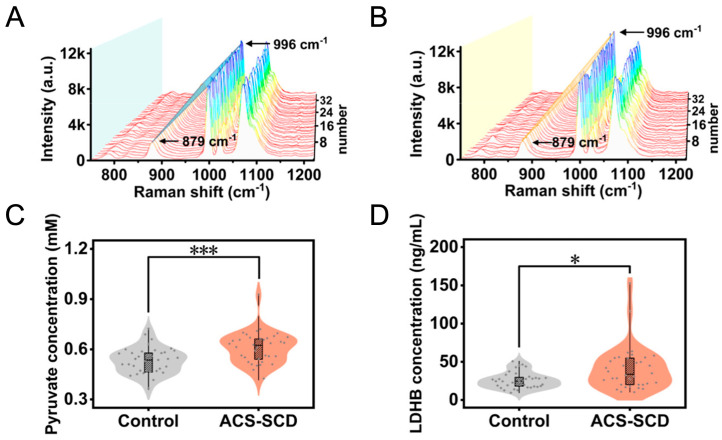
SERS spectra of plasma exosomes from (**A**) healthy controls and (**B**) ACS-SCD patients with SERS peaks arising from pyruvate (I879/I996) and LDHB (996 cm^−1^). (**C**) Pyruvate and (**D**) LDHB concentrations determined from calibration curves, with statistically different concentrations between healthy and ACS-SCD patients (*** *p* < 0.001; * *p* < 0.05). Figure adapted from Reference [119].

Acute myocardial infarction (AMI) [117] heart failure/congestive heart failure (CHF) [58], coronary artery disease (CAD)/myocardial infarction (MI) vs. CRD stratification [118], acute coronary syndrome (ACS)-sudden cardiac death (ACS-SCD) [119], and stroke/cerebrovascular injury [120] have all been studied via SERS nanotags/substrates. General strategies include nanotag-based SERS-LFIA or multiplexed assays that allow for low limits of detection (pg to fg mL^−1^), high sensitivity and high specificity (>80%) for cardiac markers, such as cardiac troponin I (cTnI); lactate dehydrogenase B (LDHB), an enzyme that converts lactate into pyruvate in the presence of oxygen in heart tissue (Figure 16); creatine kinase MB (CK-MB), a cardiac enzyme that is released with heart damage; 3-hydroxybutyrate dehydrogenase 1 (BDH1), a mitochondrial enzyme that is critical in energy metabolism and heart disease outcomes; cardiac troponin T (cTnT), a protein released in the blood due to heart damage; and aspartate aminotransferase (AST), an enzyme that is elevated with heart tissue injury (although it is also present in other organs; Table 5) [57]. Beyond cardiac markers, multiplex SERS-LFIAs and core–shell–satellite tags were used to detect matrix metalloproteinase-9 (MMP-9), an enzyme involved with breakdown of extracellular matrix, neuron-specific enolase (NSE), an enzyme found in neurons and neuroendocrine cells, N-terminal pro-B-type natriuretic peptide (NT-proBNP), a peptide fragment that is a strong biomarker in assessing ischemic and hemorrhagic stroke, and S100B, a protein found in astrocytes that is elevated in stroke, with fg–pg mL^−1^ sensitivities and serum validation [121].

### 6.5. Traumatic Brain Injury

**Figure 17:**
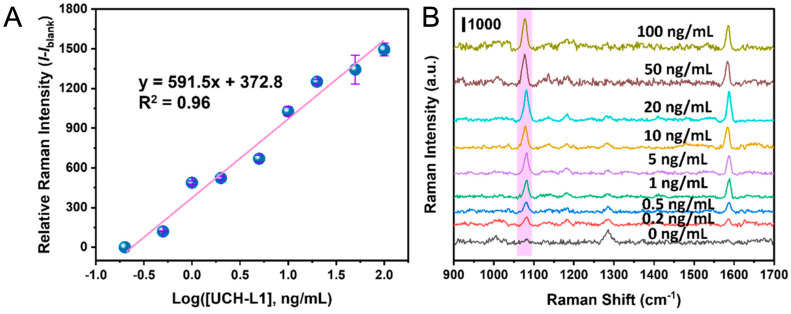
Calibration curve (**A**) and SERS spectra (**B**) of UCH-L1 in 20% plasma and 80% PBS buffer solutions on a AuNS@Ag SERS-PLFS, pink-marked section is the characteristic peak of 4-MBA (1078 cm^−1^), which works as a function of the UCH-L1 concentration [86]. Adapted from Ref. [86].

Traumatic brain injury (TBI) biomarkers, including glial fibrillary acidic protein (GFAP), a structural protein in astrocytes that is released with TBI; UCH-L1 (ubiquitin carboxyl-terminal hydrolase L1), an enzyme that is abundant in the brain and plays a role in managing protein breakdown; S-100B; myelin basic protein (MBP), a protein associated with breakdown of myelin on nerve fibers; NSE; and cytokines related to regulation of inflammation, such as interleukin-10 (IL-10) and tumor necrosis factor- α (TNF-α), have all been assayed using SERS-based nanotags. Multiple assay formats have been employed for TBI biomarker detection: paper lateral-flow SERS (SERS-PLFS; Figure 17), sandwich assays on engineered plasmonic substrates, hierarchical “tag + substrate” hot-spot architectures, and nanozyme-assisted turn-on SERS [86]. Nanotag designs span Au nanostars, Au nanorods, Au@Ag core–shells, and silica-encapsulated tags, most commonly encoded with 4-MBA/DTNB/NBA/THI and coupled via EDC/NHS or amine-reactive linkers; silica shells or polymer layers stabilize tags in plasma/whole blood [86]. Across these reports, LODs range from fg mL^−1^ (substrate-enhanced or nanozyme-amplified formats) to low ng mL^−1^ (PLFS), with SERS spectra frequently excited at 785 nm using handheld Raman systems and cross-validated against ELISA or clinical samples (Table 6).

### 6.6. Food and Drink Contaminants

**Figure 18:**
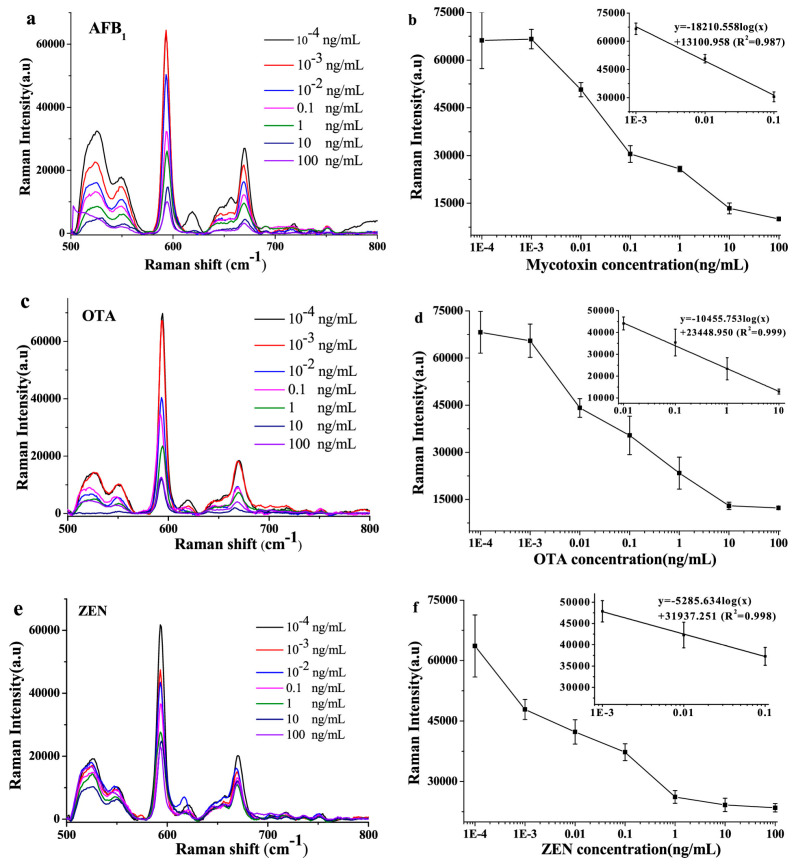
SERS spectra (**left**) and calibration curves (**right**) for quantification of (**a**,**b**) AFB1, (**c**,**d**) OTA, and (**e**,**f**) ZEN. The linear ranges used for calibration curves are shown in the insets. Adapted from Reference [75].

SERS nanotags have matured from proof-of-concept into practical, field-ready assays that can be applied for screening of illegal additives (β_2_-agonists, heavy metals, mycotoxins) with pg–fg·mL^−1^ sensitivities in food supplies. These assays can be implemented using multiplexing LFAs or vertical-flow chips and offer robust performance in real food samples (Table 7). Wu et al., 2022 [130] built a magnetic dual-readout ICA with rough Fe_3_O_4_@Au DTNB-encoded tags for the detection of clenbuterol and ractopamine, β-agonists used to increase lean muscle in meats, using a portable 785 nm Raman system and achieving LODs in the pg·mL^−1^ range. This idea was extended to a dual-channel clenbuterol + Cd^2+^ assay using raspberry-like Fe@RAu tags with embedded DTNB with pg·mL^−1^ LODs and tested in milk and pork [131]. For mycotoxins, Chen et al., 2023 [132] achieved single-T-line multiplexing detection of Aflatoxin B1 (AFB1) and Ochratoxin A (OTA), highly toxic carcinogenic molds, at sub-pg·mL^−1^ LODs in approximately 15 min in cereals with Au@SiO_2_ tags carrying orthogonal 4-MBA/DTNB reporters. Sun et al., 2021 [75] used silica photonic-crystal microspheres with AuNP@NBA immunotags to competitively screen for mycotoxins AFB1, OTA, and zearalenone (ZEN) with improved repeatability. Advancing substrates, Chen et al., 2025 [133] paired Au@Ag tags (NBA/4-MBA/DNTB) with photonic nitrocellulose inverse-opal vertical-flow chips to reach fg·mL^−1^ for OTA/AFB1/ZEN (Figure 18), and Chen et al., 2025 [134] embedded reporters in Fe_3_O_4_@Au shells for a magnetic three-plex VFA detection of mycotoxins Fumonisin B1 (FB1), AFB1, and deoxynivalenol (DON), enabling single-spot fg·mL^−1^ quantitation without preprocessing. Zhang et al., 2025 [135] introduced a membrane-like GO@PEI–AuNP tag (<3 nm gaps) plus a Fe_3_O_4_@Ag magnetic aptaprobe for a signal-off AFB1 aptasensor with pg·mL^−1^ LODs and strong HPLC concordance. Targeting another major area of contamination, other groups have produced nanotags to detect metal ions in beverage samples. Utilizing dual-reporter/targeting ligands that bind metal ions, Li et al. and Dugandžić et al. were able to detect low concentrations of mercury (II) ions in milk and copper (II) ions in white wine, respectively [48,136].

### 6.7. Alzheimer’s Disease

**Figure 19:**
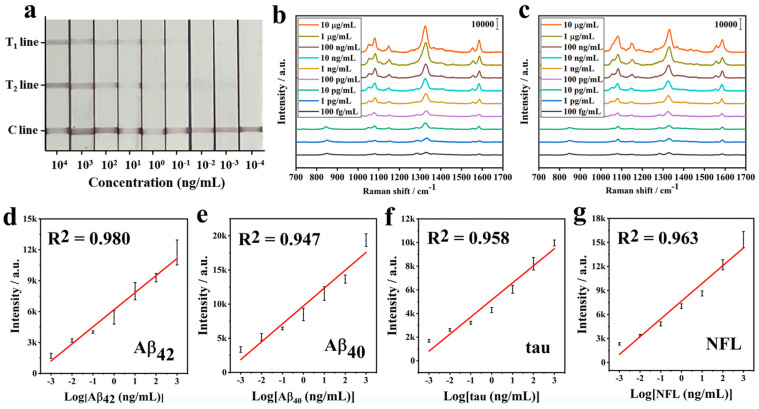
SERS-LFA for detection of AD biomarkers. (**a**) Photo of SERS-LFA test strips showing control line (C line) with two test lines (T1 line, T2 line) across a range of concentrations. SERS spectra of (**b**) line T1 and (**c**) line T2 on the test strips. Calibration curves for Raman intensities correlated with (**d**) Aβ42, (**e**) Aβ40, (**f**) tau, and (**g**) NFL. Adapted from Reference [143].

Alzheimer’s disease (AD) is a neurodegenerative disorder that leads to progressive dementia, memory loss, and cognitive decline [137,138]. It severely affects the physical and mental well-being of older adults and places a significant burden on families and society. Worldwide, more than 50 million people currently live with dementia, and this number is projected to rise to about 152 million by 2050 [139], with AD accounting for 60–70% of cases. As global populations continue to age, AD has become an increasingly serious public health and social challenge. AD is defined by two main pathological features: the buildup of amyloid-beta (Aβ) in extracellular plaques and the accumulation of abnormal tau protein [140,141]. Aβ, produced from the cleavage of amyloid precursor protein (APP), exists mainly in two peptide fragment lengths, Aβ40 and Aβ42, and becomes harmful when it aggregates, causing oxidative stress, inflammation, and synaptic dysfunction. Tau, a protein that stabilizes microtubules, becomes dysfunctional when excessively modified, contributing to neuronal degeneration. In recent years, blood-based biomarkers, such as Aβ, tau, and neurofilament light chain (NFL) [142], have shown potential for predicting AD progression, although their levels in blood are very low. Since AD is complex and variable, no single biomarker can provide a reliable early diagnosis, making sensitive multi-biomarker detection essential for accurate clinical assessment. SERS nanotags can serve as an effective tool for the specific identification and quantitative analysis of Alzheimer’s disease biomarkers (Table 8). Zhan et al. [143] developed a multiplex SERS-based lateral flow assay (SERS-LFA) using Au@SiO_2_ nanotags to simultaneously detect four major Alzheimer’s disease biomarkers—Aβ42, Aβ40, tau, and NFL. The nanotags were prepared by encoding gold core–silver shell nanoparticles with 4-MBA or DNTB as the Raman reporter molecules and coating them with a silica shell to improve stability. Antibodies were attached using epoxy-silane chemistry, allowing the formation of two sandwich immunoassays on a single test strip. The system showed high specificity, wide linear ranges (0.001–1000 ng/mL), and very low detection limits: 138.1 fg/mL for Aβ42, 191.2 fg/mL for Aβ40, 257.1 fg/mL for tau, and 309.1 fg/mL for NFL (Figure 19). These values are two orders of magnitude below typical blood concentrations, demonstrating strong potential for early AD screening in portable, low-cost formats. Similarly, Yang et al. [144] reported an ultrasensitive SERS immunosensor for tau protein detection using Cy3-labeled gold nanotags and a plasmonic head-flocked gold nanopillar substrate. By converting full IgG antibodies into half-antibody fragments with TCEP, they reduced the binding distance between nanotags and the nanopillar surface, greatly enhancing plasmonic coupling and SERS signal strength. This approach improved sensitivity by 135-fold compared to whole-antibody sensors and achieved a detection limit of 3.21 fM over a broad working range (10 fM–1 μM). The sensor also successfully measured tau levels in clinical plasma samples and clearly distinguished AD patients from healthy individuals.

### 6.8. Pregnancy and Infertility

**Figure 20:**
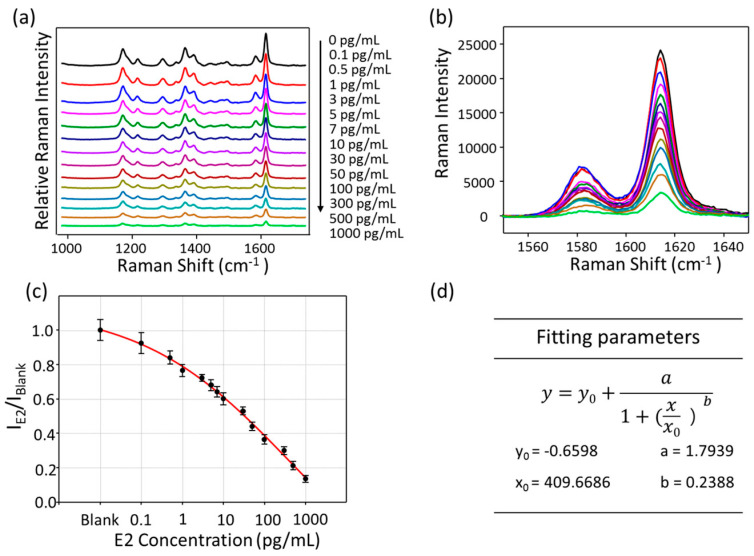
(**a**) SERS spectra for decreasing concentrations of E2; (**b**) SERS intensity variation at 1613 cm^−1^; (**c**) corresponding calibration line of the SERS signal intensity at 1613 cm^−1^ as a function of the logarithm of the concentration of E2; (**d**) four-parameter logistic function for the fitting line. Error bars indicate the standard deviation of five measurements. Adapted from Reference [153].

SERS nanotags have emerged as highly sensitive analytical platforms for detecting pregnancy and infertility-related biomarkers, due to their strong signal enhancement, molecular specificity, and compatibility with rapid point-of-care formats, such as lateral flow assays and microfluidic immunosensors (Table 9). Song et al. [148] reported an SERS-colorimetric dual-signal LFA for the simultaneous and ultrasensitive detection of PI3K and CRAF, two vital biomarkers [149,150] associated with fetal growth restriction (FGR). In this work, AgNPs embedded with the Raman reporter 4-MPA were conjugated with anti-PI3K and anti-CRAF antibodies to form SERS nanotags capable of direct analysis in unprocessed blood. Notably, the assay reached detection limits of 0.76 fg/mL (PI3K) and 0.61 fg/mL (CRAF), outperforming conventional ELISA and enabling reliable quantification in clinical samples. Here, this ultrasensitive and straightforward SERS assay provides a tool for the detection of double targets in pregnancy. Male infertility diagnosis increasingly relies on the accurate assessment of sperm-specific biomarkers, among which the intra-acrosomal protein SP10 [151] is recognized as a highly reliable indicator of sperm concentration and overall reproductive potential. Liu et al. [152] developed an ultrasensitive SERS-based lateral flow immunoassay (SERS-LFA) for quantitative detection of SP10. In this platform, gold–silver core–shell nanorod SERS nanotags embedded with DTNB Raman reporters were conjugated with anti-SP10 antibodies and integrated into a paper-based LFA device. The assay exhibited an impressive linear range from 100 fg/mL to 10 ng/mL and achieved a detection limit of 25.12 fg/mL, corresponding to only a few hundred sperm per milliliter—substantially lower than commercial colorimetric fertility strips (Figure 20). As SP10 is a key acrosomal protein released during sperm maturation, its sensitive quantification offers valuable insight into male fertility status. Overall, this SERS-nanotag-enabled LFA represents a promising, low-cost, and rapid point-of-care device for quantitative male infertility diagnostics.

### 6.9. Veterinary Drugs

**Figure 21:**
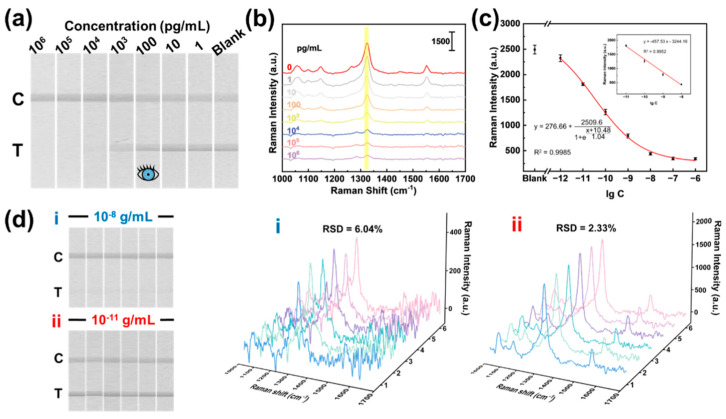
Photographs and Raman detection results of CAP. (**a**) Photograph of Au NS-based SERS−LFIA strips with different concentrations of CAP. (**b**) Average SERS spectra of T-lines corresponding to the strips (**a**). (**c**) Corresponding calibration curves of SERS intensities at 1326 cm^−1^ with concentrations of CAP. The inset shows the fitted curve of the SERS intensity vs. the lg value of the CAP concentration for a certain range. Error bars indicate the standard deviation of three measurements. (**d**) Photographs and corresponding SERS spectra of six repetitive tests of AuNS-based SERS−LFIA strips with (i) 1 × 10^−8^ and (ii) 1 × 10^−11^ g/mL of CAP, with corresponding SERS spectra and relative standard deviation (RSD) values. Adapted from Reference [157].

Veterinary antibiotics are extensively used in livestock and aquaculture to manage animal diseases, reduce infection rates, and prevent the spread of pathogens within herds and flocks [155]. While these medications play an important role in maintaining animal health and supporting agricultural productivity, their excessive or inappropriate use can have serious unintended consequences. One major concern is that these veterinary drugs can move through the food chain and ultimately reach humans. This not only reduces the effectiveness of essential antibiotics but also poses significant risks to public health and environmental stability. SERS nanotags have emerged as powerful analytical tools for rapid, ultrasensitive detection of veterinary drug residues in food and animal-derived samples (Table 10). Liu et al. [156] developed an ultrasensitive multiplex magnetic–SERS immunoassay for simultaneous quantification of the veterinary antibiotics chloramphenicol (CAP) and tetracycline (TTC). The system employs gold–silver core–shell nanostar nanotags (AuNS@Ag) in which Raman reporters (4-MBA or DTNB) are embedded within the nanogap region created during Ag shell overgrowth, producing strong plasmonic hotspots and highly stable spectral signatures. These nanotags are paired with monoclonal anti-antibiotic antibodies immobilized on Fe_3_O_4_ magnetic nanoparticles, allowing rapid target extraction. The assay achieves remarkably low detection limits 159.49 fg/mL for CAP and 294.12 fg/mL for TTC—with broad linear ranges from 10 pg/mL to 1 μg/mL (Figure 21). Application to whole-milk samples demonstrates excellent accuracy (92–110% recovery) and strong linear correlations, underscoring the robustness of this platform for real-world food-safety surveillance.

### 6.10. Water Contamination Monitoring

There is increasing concern regarding contamination of the water supply and environment by pesticides, heavy metals, and plastics. These threats make large-scale and efficient water monitoring methods essential [161,162]. Pesticides are widely used to maintain agricultural productivity by controlling pests, weeds, and disease vectors. However, excessive or improper application has led to residue accumulation in the water supply that poses serious environmental and public health risks [163]. Similarly, heavy metal ions and microplastics entering the water supply from various sources pose similar threats to public health and safety. To address these contaminants, SERS nanotags have emerged as powerful analytical tools, offering high sensitivity, strong specificity, and rapid on-site detection capabilities for water and runoff monitoring (Table 11). To detect pesticides, Sheng et al. [164] developed a multiplex SERS-LFA test strip for the simultaneous detection of three widely used pesticides—chlorothalonil (CHL), imidacloprid (IMI), and oxyfluorfen (OXY). For this application, a nanotag was created using 4-nitrothiophenol (4-NTP), which was embedded between a silver core and gold shell, as the Raman reporter. A complete SERS-LFA setup was created by utilizing ssDNA–streptavidin to create a dual-binding interaction with nanotags, effectively boosting the overall detection signal to achieve ultralow detection limits with high sensitivity in real samples. Santhoshkumar et al. developed a AgNP/GO/g-CN nanohybrid to detect potentially dangerous levels of mercury (II) (Hg^2+^) ions at ppm levels in aqueous solutions, demonstrating that rationally designed SERS substrates can be used to monitor wastewater for metal ion contamination [165]. Kumar et al. developed a toluene sample dispersion combined with SERS on filter paper to detect various types of microplastics from lake and salt water samples [166].

## 7. Conclusions

This review seeks to summarize SERS nanotags and their use as sensitive, versatile, and customizable diagnostic sensors. The three main components of the nanotag, the plasmonic core, the Raman reporter molecule, and the targeting ligand, must be carefully integrated to achieve optimal performance in biomedical sensing applications. Successful nanotag development requires thorough consideration of multiple interdependent factors, including the selection of plasmonic materials and geometries for maximum enhancement, the choice of reporter molecules with distinct spectral signatures for multiplexing capabilities, and the functionalization strategies that ensure stability and sensitivity in complex biological environments.

Recent advances in SERS nanotags include using machine learning and computational modeling to create nanostructures with controlled optical properties and to optimize assay conditions with unprecedented precision. In addition, the integration of SERS nanotags into practical diagnostic platforms, particularly lateral flow assays and microfluidic devices, demonstrates their potential to bridge the gap between laboratory research and clinical point-of-care testing. Despite significant progress, several challenges remain. Standardization of nanotag synthesis and characterization protocols is needed to improve the reproducibility of SERS nanotags across laboratories. Also, long-term stability of SERS nanotags and the scalability of production are hurdles still to be fully addressed.

Looking forward, SERS nanotags are poised to play an increasingly important role in a wide range of biomedical fields. Bringing together spectroscopy and nanotechnology has created opportunities for developing next-generation diagnostic tools that are not only highly sensitive and specific but also accessible and affordable. As this field continues to mature, collaborative efforts between chemists, biologists, clinicians, and engineers will be essential to fully realize the potential of SERS nanotags and to address the growing demand for rapid, multiplexed diagnostic solutions in healthcare and many other fields.

## Figures and Tables

**Table 1:** Table of common RRMs, the IUPAC names, and respective structures.

Common Name	IUPAC Name	Structure
DTNB	5,5′-dithiobis(2-nitrobenzoic acid)	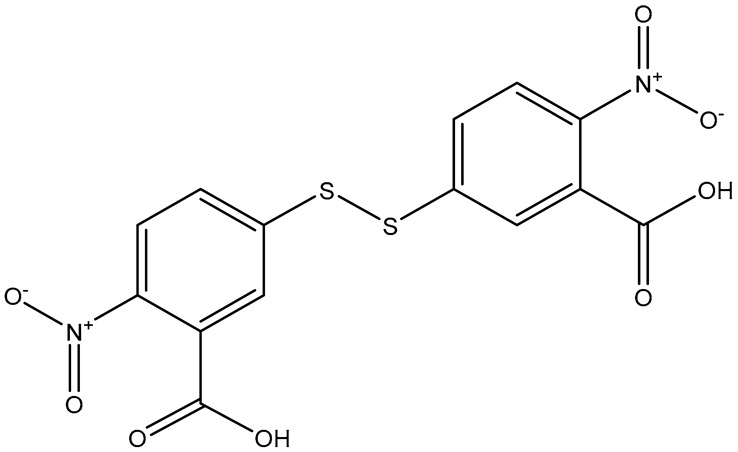
MGITC	malachite green isothiocyanate	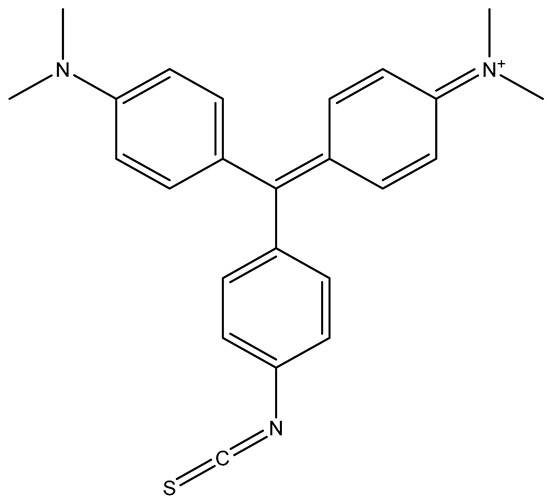
4-MBA	4-mercaptobenzoic acid	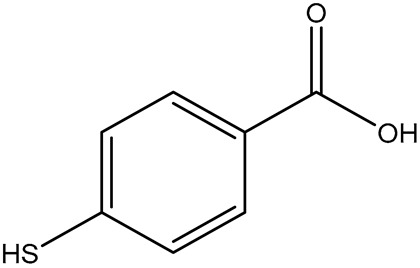
TFMBA	tetrafluoro-4-mercaptobenzoic acid	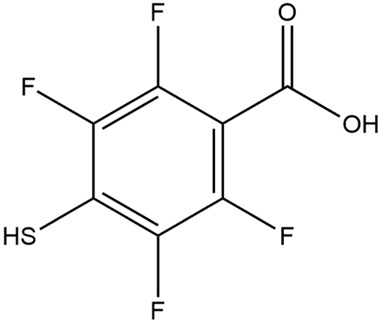
2-MPY	2-mercaptopyridine	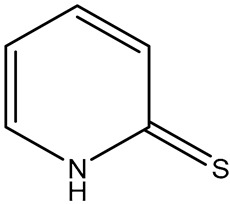
DSNB	5,5′-dithiobis(succinimidyl-2-nitrobenzoate)	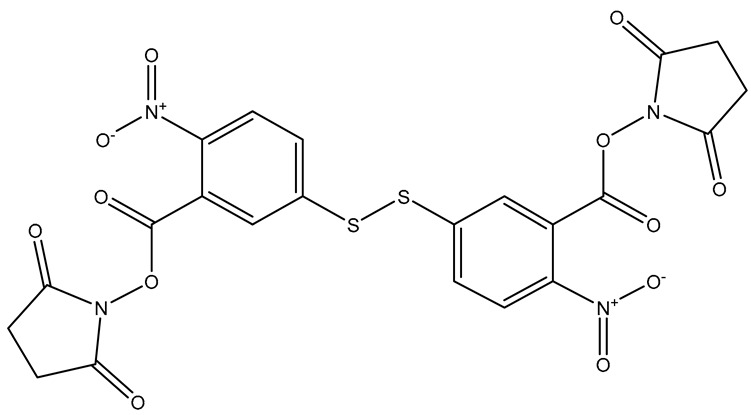
HITC	hexamethylindotricarbocyanine iodide	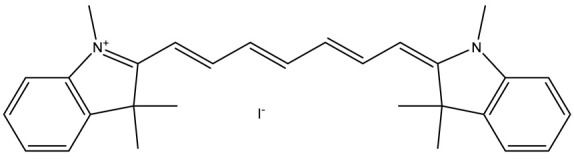
Cy5	Cyanine 5	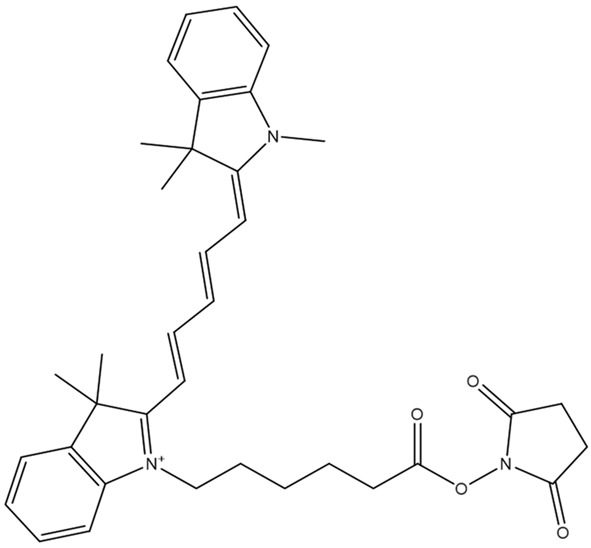
4-NTP	4-nitrothiophenol	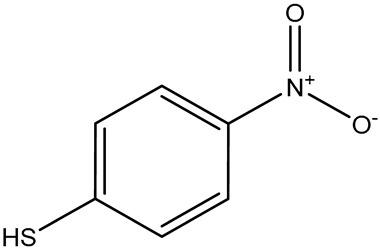
NBA/NB	Nile Blue A/Nile blue	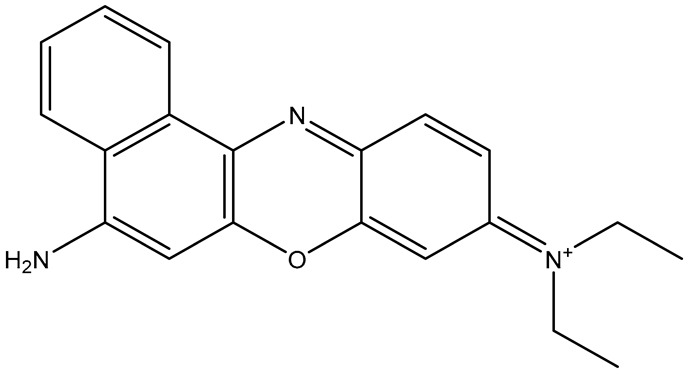
R6G	Rhodamine 6G	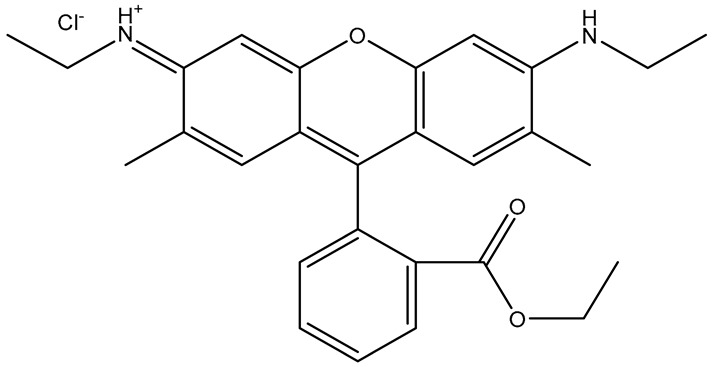
4-ATP/p-ATP	4-aminothiophenol	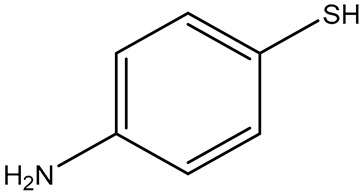

**Table 2:** Applications of SERS nanotags in detection of bacteria.

Bacterial Species	Detection Target	Sensor Application	Targeting Ligand	Plasmonic Core Type	Reporter	Ligand Conjugation	Coating	LOD	Citation
*M. tuberculosis*	ManLam	Gold capture immunoassay	Antibody	AuNPs	DSNB	Reporter linked	BSA	1.1 ng/mL	[22]
*E. coli*, *S. typhimurium*, and *S. aureus*	Bacterial Cell Wall	Magnetic isolation in solution	Antibody	Ag@Fe_3_O_4_	MGTIC, DACITC, PPY	EDC/NHS	PEG	10 CFU/mL (all)	[24]
*E. coli*	Bacterial Cell wall	Magnetic isolation in solution	Aptamer	Magnetic beads-AuNPs	Cy5	Thiol conjugation	BSA	5.9 × 10^3^ CFU/mL	[97]
Group A *S. pyrogenes*	Bacterial cell wall	LFA	Antibody	Fe_3_O_4_@AuNPs and AuNRs	DTNB	EDC/NHS and Strepadvidn/Biotin	BSA	0.2 CFU/mL	[98]
*P. gingivalis*	LPS	LFA	Antibody	Ag@AuNSs	HITC	Direct	BSA	<10 ng/mL	[99]
*L. monocytogenes*, *C. jejuni*, and *S. aureus*	Bacterial Cell Wall	Magnetic LFA	Wheat germ agglutinin (WGA)	Fe_3_O_4_@AuNP	DTNB	EDC/NHS	PVP	10 cells/mL	[100]
*C. difficile*	SplA and ToxB	LFA	Antibody	AuNP	MGTIC	Direct	BSA	0.01 pg/µL	[101]
*S. Typhimurium*	Bacterial cell wall	Solution	Aptamer	Ag@AuNRs	4-MBA	Direct	None	64 CFU/mL	[102]
*E. coli* (O157:H7)	Bacterial Cell wall	Microfluidic assay	Antibody	AuNPs	R6G	EDC/NHS and Strepadvidn/Biotin	None	0.5 CFU/mL	[103]
*S. aureus*	Bacterial Cell Wall	Magnetic isolation in solution	Aptamer	Au@Fe_3_O_4_	MBA	Direct (low temperature)	None	6.91 CFU/mL	[104]

**Table 3:** Applications of SERS nanotags for detection of viruses.

Viral Species Target	Target Analyte	Application	Targeting Ligand	Plasmonic Core Type	Reporter	Ligand Conjugation	Coating	LOD	Citation
Dengue	Dengue NS1 protein	Microelectrode assay	Antibody	Au/Ag Nanoboxes	MMC	EDC/NHS	BSA	10 pg/mL	[63]
SARS-Cov-2	Anti SARS-Cov-2 antibodies	LFA	Recombinant nucleocapsid protein	Gap enhanced AuNPs	4-NBT	Direct	BSA	1 ng/mL (IgM) 0.1 ng/mL (IgG)	[88]
SARS-Cov-2	nucleoprotein	Magnetic microdroplet	Antibody	Au@CoNPs	MGITC	EDC/NHS	PEG	0.22 PFU/mL	[105]
Influenza A	Anti-Influenza A antibodies	LFA	Antibody	Ag@Au Nanostars	4-MBA	Direct	Milk powder	8.0 pg/mL	[106]
Human papillomavirus (HPV)	Viral DNA	solution	CRISPR/dCas9/sgRNA	AuNS@SiO_2_	oxTMB	n/a	PEI	30 ng	[107]
Zika	Zika NS1 protein	LFA	Antibody	AuNP@SiO_2_	NBA	EDC/NHS	Silica	10 ng/mL	[108]
Monkeypox	Viral particle	LFA	Antibody	AuNPs	IR808	EDC/NHS	none	1 pg/mL	[109]
Tobacco Mosaic Virus	Viral RNA	Hydrogel	Aptamer	Ag@Au Nanostars	Cy5	Direct	PEG	n/a	[110]
Porcine epidemic diarrhea virus and porcine group A rotavirus	Viral particle	LFA	Antibody	Au@AgNPs	4-MBA	Direct	BSA	80.1 TCID_50_/ mL (PEDV) 319 copies/μL (PoRVA)	[111]
Hepatitis C	Viral RNA	Solution	Aptamer	AuNPs	MGTIC	pH assisted direct	none	1.706 fM	[112]

**Table 4:** Applications of SERS nanotags for cancer diagnostics.

Cancer Type	Target	Application	Ab/Aptamer	Nanostructure	Reporter	Conjugation	Coating	LOD	Citation
Lung	m-SHOX2	SERS immunoassay	m-SHOX2 DNA	Au@AgNP	MBA	DSP	dATP	0.52 pM	[35]
	m-RASSF1A		m-RASSF1A DNA		TFMBA			0.66 pM	
Mealona, breast, lung	CD63	ESCP microarray SERS barcodes	Anti-CD63	AuNPs	MBA, TFMBA, DTNB, MPY	DTSSP	BSA	10^3^ sEVs mL^−1^ for	[50]
	CD9		Anti-CD9						
	CD81		Anti-CD81						
	MCSP		Anti-MCSP						
	MCAM		Anti-MCAM						
	ErbB3		Anti-ErbB3						
	LNGFR		Anti-LNGFR						
Osteosarcoma	CD63	Micro-fluidic SERS	Anti-CD63	AuNP	4-MBA	EDC/NHS	BSA	~2 exosomes µL^−1^	[51]
	CD9		Anti-CD9		TFMBA				
	EpCAM		Anti-EpCAM		4-MPY				
Breast	HER2	Liquid-based bead assay	HER2 aptamer	AuNP	DTNB	Direct	BSA	~32 exosomes µL^−1^	[52]
Colorectal	CEA		CEA aptamer		MMC			~73 exosomes µL^−1^	
Prostate	LNCaP	Liquid-based bead assay	PSMA aptamer	Au@Ag nanotrepang	2-MPY	Direct	BSA	26 exsomes µL^−1^	[53]
Breast	SKBR3		HER2 aptamer		4-ATP			72 omes µL^−1^	
Liver	HepG2		AFP aptamer		NTP			35 omes µL^−1^	
Pancreatic	GPC1	Liquid-based bead assay	Anti-GPC1	AuNP	DTNB	DTSSP	BSA	2.3 × 10^6^ particles/mL	[54]
	EpCAM		Anti-EpCAM		4-MBA				
	CD44v6		Anti-CD44v6		TFMBA				
Breast	BRCA1	SERS chip	BRCA1 DNA	Au@AgNP	R6G	Direct	BSA	~0.61 pM	[55]
	BRAC2		BRCA2 DNA		4-MBA			~0.78 pM	
Pancreatic	MIF	Array-based assay	Anti-MIF	Au@AgNP	p-ATP	Direct	BSA	1 exosome in 2 µL	[68]
	GPC1		Anti-GPC1						
	EGFR		Anti-EGFR						
Ovarian	PLAP	SERS Sandwich immunoassay	Anti-PLAP	Mesoporus Au	4-MBA	DSP	BSA	10^2^ EVs mL^−1^	[70]
Multiple Myeloma	CD138	SERS assay	Anti-CD138	AuNP	MBA	DSP	BSA	1 cell mL^−1^	[71]
	CD38		Anti-CD38		TFMBA				
Breast	CD9	ADSP-SERS assay	Anti-CD9	AuNPs	DTNB	DTSSP	ADSP layer	-	[72]
	EpCAM		Anti-EpCAM		4-MBA				
	HER2		Anti-HER2		TSA				
Pancreatic, breast	EGFR	SERS imaging	VMC10 DNAzyme	AgNPs	p-ATP	EDC/NHS	PEG	1.48 pM	[73]
	c-Myc								
	H19								
Breast	CD63	Micro-pillar array	CD63 aptamer	AuNPs	DTNB	Direct	PEG	2.11 particles µL^−1^	[74]
	EpCAM		EpCAM aptamer		4-MBN			1.17 particles µL^−1^	
Lung	CD63	Micro-fluidic chip	Anti-CD63	AuNP	MBA	EDC/NHS	BSA	5.46 × 10^3^ particles/mL	[77]
	PD-L1		Anti-PD-L1		DTNB			2.44 × 10^3^ particles/mL	
	EGFR		Anti-EGFR		TFMBA			4.46 × 10^2^ particles/mL	
Breast	HER2	SERS-LFA	HER2 aptamer	AuNS	DTNB	EDC/NHS	BSA	3.27 × 10^6^ particles/mL	[78]
	MUC1		MUC1 aptamer		4-MBA			4.80 × 10^6^ particles/mL	
Various Cancers	IL-6	SERS sandwich immunoassay	Anti-IL-6	Au@AgNP	NBA	APTES	BSA	12.4 fg/mL	[79]
Prostate	t-PSA	SERS sandwich immunoassay	Anti-PSA	Ag-Au nanoflowers	4-MBA	EDC/NHS	BSA	100 fg mL^−1^	[80]
Ovarian	CD81	Microfluidic SERS chip	Anti-CD81	Au@AgNP	MGITC	EDC/NHS	BSA	10 particles mL^−1^	[81]
	CD9		Anti-CD9						
	EpCAM		Anti-EpCAM						
	EGFR		Anti-EGFR						
	CD24		Anti-CD24						
	CA125		Anti-CA125						
Colon	Clusterin	Sandwich SERS Simmunoassay	Anti-Clusterin	AuNPs	4-NBT	Direct	BSA	3.0 ng mL^−1^	[82]
Ovarian	CA125	Sandwich SERS immunoassay	Anti-CA125	AuNR	4-MBA	Direct	BSA	1 U mL^−1^	[83]
Lung	Cas12a	SERS assay	crRNA-Cas12a	AuNP	TFMBA	Biotin-ssDNA	Streptavidin	0.62 aM	[84]
Ovarian	MUC16	Magnetic SERS	Anti-MUC16	AuNPs	4-MBA	EDC/NHS	BSA	2.13 EVs µL^−1^	[85]
	CLDN3		Anti-CLDN3		DTNB				
	FOLR1		Anti-FOLR1		TFMBA				
Pancreatic	EpCAM	Array based-assay	Anti-EpCAM	Au@AgNP	4-NTP	Direct	Graphene oxide	2.7 × 10^2^–0.7 × 10^8^ particles mL^−1^	[87]
Breast	HER2		Anti-HER2						
Prostate	PSA	Sandwich assay	Anti-PSA	AuNP	IR808	EDC/NHS	BSA	0.029 ng mL^−1^	[113]
Prostate	Prion protein	Microfluidic assay	Anti-Prion protein	AuNP	MGITC	Direct	BSA	Single cell	[114]
	PSMA		Anti-PSMA		Nile blue				
Gastric	EFA1	SERS CHA	Anti-EFA1	Au@Pt nanozyme	Ox-TMB	Direct	BSA	0.75 pg mL^−1^	[115]
	MMP 13		Anti-MMP 13					0.84 pg mL^−1^	
Lung	CEA	Paper-based assay	Anti-CEA	AuNPs	MBA	EDC/NHS	Wax	0.92 pg mL^−1^	[116]
	NSE		Anti-NSE		DTNB			1.56 pg mL^−1^	

**Table 5:** Applications of SERS nanotags for cardiovascular diagnostics.

Type	Target	Application	Ab/Aptamer	Nanostructure	Reporter	Conjugation	Coating	LOD	Citation
Stroke	NSE	SERS-LFA	Anti-NSE	Au@AgNP	NBA	Direct	BSA	0.53 pg mL^−1^	[56]
	S-100β		Anti-S-100β		4-MBA			0.35 pg mL^−1^	
AMI	cTnI	SERS-LFIA	Anti-cTnI	Au@Au	NBT	Direct	Silica	0.65 pg mL^−1^	[57]
Heart failure	cTnI	Multiplex SERS assay	cTnI aptamer	Au@AgNP	2-MPY	EDC/NHS	BSA	0.1 pg mL^−1^	[58]
	sST2		Anti-sST2		4-NTP			1.0 fg mL^−1^	
	NT-proBNP		NT-proBNP aptamer		2-NT			1.0 fg mL^−1^	
AMI	CK-MB	SERS chip	Anti-CK-MB	AuNPs	4-MBA	Direct	BSA	6.56 fg mL^−1^	[59]
	cTnI		Anti-cTnI		2-NAT			11.81 fg mL^−1^	
AMI	cTnI	SERS immunoassay	Anti-cTnI	AuNP	MGITC	EDC/NHS	BSA	5.8 pg mL^−1^	[117]
CAD/MI vs. CRD	cTnI	Multiplex SERS	Anti-cTnI	Au-Ag nanobox	MMC	EDC/NHS	BSA	0.205 pg mL^−1^	[118]
	CK-MB		Anti-CK-MB		MBN			8.91 pg mL^−1^	
	LDHB		Anti-LDHB		DTNB			0.00744 pg mL^−1^	
	BDH1		Anti-BDH1		MMTAA			0.024 pg mL^−1^	
	cTnT		Anti-cTnT		TSA			0.367 pg mL^−1^	
	AST		Anti-AST		MPY			9.44 pg mL^−1^	
ACS-SCD	LDHB	Gemini SERS	Anti-LDHB	AgNPs	3-MPBA	EDC/NHS	BSA	0.032 ng mL^−1^	[119]
Stroke	MMP-9	SERS-LFIA	Anti-MMP-9	Au@AgNP	DTNB	Direct	BSA	0.00020 ng mL^−1^	[120]
	NSE		Anti-NSE					0.00016 ng mL^−1^	
	NT-proBNP		Anti-NTproBNP					0.00012 ng mL^−1^	
Stroke	S-100β	Sandwich Assay	Anti-S-100β	AuAg nanobox	4-NTP	EDC/NHS	BSA	1.7 fg mL^−1^	[121]
AMI	cTnI	SERS-LFIA	Anti-cTnI	Au@PS	4-ATP	Direct	BSA	~1 pg mL^−1^	[122]
	NT-proBNP		Anti-NT-proBNP					~10 pg mL^−1^	
AMI/ACI	cMyBP-C	SERS-LFIA	Anti-cMyBP-C	Au@AgNP	4-NBT	Direct	BSA	0.77 pg mL^−1^	[123]
AMI	cTnI	Microfluidic SERS immunoassay	Anti-cTnI	AuNP	MGITC	Direct	BSA	5.04 pg mL^−1^	[124]
	CK-MB		Anti-CK-MB		NB			2.34 pg mL^−1^	
AMI	cTnI	SERS-LFIA	Anti-cTnI	AuNP	Ru(bpy)_3_]^2+^	Direct	BSA	60 pg mL^−1^	[125]
AMI	cTnI	SERS immunoassay	Anti-cTnI	AuNP	4-MP	Direct	BSA	0.33 pg mL^−1^	[126]

**Table 6:** Applications of SERS nanotags for traumatic brain injury diagnostics.

Target	Application	Ab/Aptamer	Nanostructure	Reporter	Conjugation	Coating	LOD	Citation
GFAP	SERS-PLFS	Anti-GFAP	AuNRs	THI	EDC/NHS	BSA	0.78 pg/mL	[36]
MBP		Anti-MBP					0.91 pg/mL	
NSE	SERS-FGH	Anti-NSE	Au nanocage	DTNB	EDC/NHS	BSA	0.36 ng/mL	[37]
GFAP	SERS immunoassay	Anti-GFAP	AuNR	NBA	EDC/NHS	BSA	0.018 pg/mL	[60]
TNF- α		Anti- TNF- α		4-MPBA			0.023 pg/mL	
S-100β	SERS Sensor	Anti-S-100β	AuNR	NBA	EDC/NHS	BSA	4.57 x10^−2^ fg/mL	[61]
IL-10		Anti-IL-10		DTNB			7.84 x10^−2^ fg/mL	
UCH-L1	SERS-PLFS	Anti-UCH-L1	AuNS@Ag	4-MBA	EDC/NHS	Silica	0.08 ng/mL	[86]
S-100β	SERS-PLFS	Anti-S-100β	AuNS	4-MBA	EDC/NHS	BSA	5 pg/mL	[127]
GFAP	SERS-immunoassay	Anti-GFAP	AuNS	DTNB	EDC/NHS	BSA	1 pg/mL	[128]
S-100β	SERS immunoassay	Anti-S-100β	MoO_3−x_/CuS nanozyme	MG	EDC/NHS	Chitosan	0.47 pg/mL	[129]

**Table 7:** Applications of SERS nanotags for food and drink safety.

Type	Target	Application	Ab/Aptamer	Nanostructure	Reporter	Conjugation	Coating	LOD	Citation
Metal ion contamination	Mecury (II) ions	In-solution assay	Ion-capturing ligand	AuNS@Ag Nanocubes	Rhodamine B (Reporter also acts as targeting ligand)	Toluene-based Ag-N	None	5.16 ppb	[48]
Food safety mycotoxins	AFB1	SERS-immunoassay	Anti-AFB1	AuNPs	NBA	EDC/NHS	PEG, BSA	0.82 pg mL^−1^	[75]
	OTA		Anti-OTA					1.43 pg mL^−1^	
	ZEN		Anti-ZEN					1.00 pg mL^−1^	
Illegal veterinary drug	CLE	Magnetic SERS	Anti-CLE	Fe_3_O_4_@Au	DTNB	EDC/NHS	BSA	7.8 pg mL^−1^	[130]
	RAC		Anti-RAC					3.5 pg mL^−1^	
Food safety	CLE	Magnetic SERS chip	Anti-CLE	Fe@Au	DTNB	EDC/NHS	BSA	0.48 pg mL^−1^	[131]
	Cd^2+^		Anti-Cd^2+^-EDTA					1.88 pg mL^−1^	
Food safety mycotoxins	AFB1	SERS-LFA	Anti-AFB1	AuNPs	4-MBA	EDC/NHS	SiO_2_	0.24 pg mL^−1^	[132]
	OTA		Anti-OTA		DTNB			0.37 pg mL^−1^	
Food safety mycotoxins	OTA	SERS-VFA	Anti-OTA	Au@AgNPs	NBA	Direct	BSA	8.2 fg mL^−1^	[133]
	AFB1		Anti-AFB1		4-MBA			13.7 fg mL^−1^	
	ZEN		Anti-ZEN		DTNB			47.6 fg mL^−1^	
Food safety mycotoxins	FB1	SERS VFA	Anti-FB1	Fe_3_O_4_@Au	NBA	Direct	BSA	0.053pg mL^−1^	[134]
	AFB1		Anti-AFB1		4-MBA			0.028 pg mL^−1^	
	DON		Anti-DON		DNTB			0.079 pg mL^−1^	
Food safety mycotoxins	AFB1	SERS Aptasensor	AFB1 aptamer	AuNPs	DTNB	EDC/NHS	BSA	2.31 pg mL^−1^	[135]
Metal ion contamination	Copper (II) ions	In-solution assay	Ion-capturing ligand	AuNPs	(Reporter also acts as targeting ligand	Thiol-gold	None	3.5 x10^−8^ M	[136]

**Table 8:** Applications of SERS nanotags for Alzheimer’s disease diagnostics.

Target	Application	Ab/Aptamer	Nanostructure	Reporter	Conjugation	Coating	LOD	Citation
Aβ42	SERS-LFA	Anti-Aβ42 monoclonal antibody	Au@SiO_2_	4-MBA	GPTMES	BSA	138.1 fg/mL	[143]
Aβ40		Anti-Aβ40 monoclonal antibody	Au@SiO_2_	DTNB	GPTMES	BSA	191.2 fg/mL	
Tau		Anti-tau monoclonal antibody	Au@SiO_2_	4-MBA	GPTMES	BSA	257.1 fg/mL	
NFL		Anti-NFL monoclonal antibody	Au@SiO_2_	DTNB	GPTMES	BSA	309.1 fg/mL	
Tau	Sandwich SERS immunosensor	Tau antibody	Au nanopillar	Cy3	Direct	BSA	3.21 fM	[144]
Aβ1-42	Sandwich SERS immunoassay (magnetic)	Anti-Aβ antibody	Tannin-capped AgNPs	4-MBA	EDC/NHS	BSA	1.62 fg/mL	[145]
P-Tau-181		Anti-Tau-181 antibody	Tannin-capped AgNPs	4-MBA	EDC/NHS	BSA	5.74 fg/mL	
p-tau^396,404^	SERS-LFA	Anti 3G5 antibody	AuNP	4-MBA	Direct	BSA	3.8 pg/mL	[146]
NfL	SERS-LFA	Anti-NfL antibody	AuNR@MQD	4-ATPP	EDC/NHS	BSA	17.38 pg/mL	[147]

**Table 9:** Applications of SERS nanotags for monitoring reproductive health.

Target	Application	Ab/Aptamer	Nanostructure	Reporter	Conjugation	Coating	LOD	Citation
PI3K	SERS-LFA	Anti-PI3K antibody	AgNPs	4-MPA	EDC/NHS	BSA	0.76 fg/mL	[148]
CRAF		Anti-CRAF antibody	AgNPs	4-MPA	EDC/NHS	BSA	0.61 fg/mL	
SP10	SERS-LFA	Anti-SP10 monoclonal antibody	AuNR@Ag	DTNB	Direct	BSA	25.12 fg/mL	[152]
17B-estradiol	SERS-LFA	Anti-E2 monoclonal antibody	AuNPs	MGITC	EDC/NHS	PBS	PEGylation	[153]
TSH	SERS-LFA	Anti-TSH antibody	AuNPs	MGITC	Direct	BSA	0.025 µIU/mL	[154]

**Table 10:** Applications of SERS nanotags for detection of veterinary drugs.

Target	Application	Ab/Aptamer	Nanostructure	Reporter	Conjugation	Coating	LOD	Citation
Chloramphenicol (CAP)	Magnetic SERS immunoassay	Anti-CAP monoclonal antibody	AuNS@Ag	4-MBA	EDC/NHS	BSA	159.49 fg/mL	[156]
Tetracycline (TTC)	Magnetic SERS im-munoassay	Anti-TTC monoclonal antibody	AuNS@Ag	DTNB	EDC/NHS	BSA	294.12 fg/mL	
Chloramphenicol (CAP)	SERS-LFA	Anti-CAP monoclonal antibody	AuNS	DTNB	Direct	BSA	1.6 pg/mL	[157]
Methamphetamine (MET)	SERS immunoassay	MET antibody	AuNPs	4-MBA	EDC/NHS	-	0.1 ng/mL	[158]
Ketamine (KET)		KET antibody	AuNPs	4-MBA	EDC/NHS	-	1 ng/mL	
Morphine (MOP)		MOP antibody	AuNPs	4-MBA	EDC/NHS	-	1 ng/mL	
Streptomycin (STR)	SERS–Vertical flow assay	Anti-STR monoclonal antibody	AuNC@Ag	DTNB	Direct	BSA	29.1 fg/mL	[159]
Avermectin (AVM		Anti-AVM monoclonal antibody	AuNC@Ag	4-MBA	Direct	BSA	38.9 fg/mL	
Kanamycin (KAN)	Magnetic SERS-LFA	Anti-KAN monoclonal antibody	Fe_3_O_4_ core + Au@Ag	DTNB	EDC/NHS	BSA	0.52 pg/mL	[160]
Ractopamine (RAC)		Anti-RAC monoclonal antibody	Fe_3_O_4_ core + Au@Ag	DTNB	EDC/NHS	BSA	2.5 pg/mL	
Chloramphenicol (CAP)		Anti-CAP monoclonal antibody	Fe_3_O_4_ core + Au@Ag	4-MBA	EDC/NHS	BSA	0.87 pg/mL	
Clenbuterol hydrochloride (CLE)		Anti-CLE monoclonal antibody	Fe_3_O_4_ core + Au@Ag	4-MBA	EDC/NHS	BSA	6.2 pg/mL	

**Table 11:** Applications of SERS nanotags for water contamination monitoring.

Target	Application	Ab/Aptamer	Nanostructure	Reporter	Conjugation	Coating	LOD	Citation
Chlorothalonil (CHL)	SERS-LFA	Anti-CHL antibody	Ag@AuNPs	4-NTP	EDC/NHS	BSA/Tween-20/sucrose	0.22 ng/mL	[164]
Imidacloprid (IMI)		Anti-IMI antibody	Ag@AuNPs	4-NTP	EDC/NHS	BSA/Tween-20/sucrose	0.28 ng/mL	
Oxyfluorfen (OXY)		Anti-OXY antibody	Ag@AuNPs	4-NTP	EDC/NHS	BSA/Tween-20/sucrose	0.34 ng/mL	
Mercury (II) ions	In Solution Assay	MB	AgNPs/GO/g-CN	MB (reporter is same as targeting ligand)	Thiol-silver	None	0.01986 ppm	[165]
Micoplastics (PVC, PP, PS)	Solution on filter paper	Thiophenol	AgNPs@filter paper	Thiophenol	Thiol-silver	None	0.001 mg/mL	[166]
Imidacloprid (IMI)	SERS-LFA	Anti-IMI monoclonal antibody	Au@Ag	4-MBA	Direct	BSA	8.6 pg/mL	[167]
Pyraclostrobin (PYR)		Anti-PYR monoclonal antibody	Au@Ag	4-MBA	Direct	BSA	97.4 pg/mL	
Aflatoxin B1 (AFB1)		Anti-AFB1 monoclonal antibody	Au@Ag	4-MBA	Direct	BSA	8.9 pg/mL	
Carbendazim (CBZ)	SERS-LFA	Anti-CBZ antibody (Ab1)	AuNPs	PB (prussian blue)	Direct	BSA	0.03 ng/mL	[168]
Imidacloprid (IMI)		Anti-IMI antibody (Ab2)	Au@Au NP	4-MBN	Direct	BSA	0.04 ng/mL	
Fipronil	SERS-LFA	Anti mouse kappa light chain	Au@Ag@Ag	DTNB	Direct	BSA	4.6 pg/mL	[169]

## Data Availability

No new data were created or analyzed in this study. Data sharing is not applicable to this article.

## Abbreviations

The following abbreviations are used in this manuscript:

2-MPY	2-Mercaptopyridine
2-NAT	2-Naphthalenethiol
2-NT	2-Nitrothiophenol
3-MPBA	3-Mercaptophenylboronic acid
4-ATP	4-Aminothiophenol (para-aminothiophenol)
4-MBA	4-Mercaptobenzoic acid
4-MBN	4-Mercaptobenzonitrile
4-MP	4-Mercaptophenol
4-MPA	4-Mercaptopropionic acid
4-MPBA	4-Mercaptophenylboronic acid
4-MPY	4-Mercaptopyridine
4-NBT	4-Nitrobenzenethiol (4-nitrothiophenol)
4-NTP	4-Nitrothiophenol (para-nitrothiophenol)
ACS	Acute coronary syndrome
ACS-SCD	Acute coronary syndrome–sudden cardiac death
AD	Alzheimer’s disease
AFP	Alpha-fetoprotein
AFB1	Aflatoxin B1
Ag	Silver
AgNPs	Silver nanoparticles
AMI	Acute myocardial infarction
APP	Amyloid precursor protein
APTES	(3-Aminopropyl)triethoxysilane
AST	Aminotransferase
Au	Gold
Au@Ag NPs	Gold core–silver shell nanoparticles
AuNPs	Gold nanoparticles
AuNS	Gold nanostars
Aβ	Amyloid-beta
BDH1	3-Hydroxybutyrate dehydrogenase 1
BRCA1	Breast cancer gene 1
BRCA2	Breast cancer gene 2
BPE	1,2-Bis(4-pyridyl)ethylene
BSA	Bovine serum albumin
CA125	Cancer antigen 125
CAD	Coronary artery disease
CAP	Chloramphenicol
Cas12a	CRISPR-associated protein 12a
CEA	Carcinoembryonic antigen
CHF	Congestive heart failure
CK-MB	Creatine kinase MB
CLDN3	Claudin-3
cTnT	Cardiac troponin T
CRISPR-Cas9	Clustered regularly interspaced short palindromic repeats–Cas9-based assay
Cy3	Cyanine 3
Cy5	Cyanine 5
DACITC	Diacetyl indocarbocyanine isothiocyanate
DCT	3,5-Dichlorobenzenethiol
DON	Deoxynivalenol
DSP	3,3′-Dithiobis(succinimidyl propionate)
DSNB	Disulfide nitrobenzoic acid derivative
DTNB	5,5′-Dithiobis(2-nitrobenzoic acid)
DTSP	3,3′-Dithiobis(sulfosuccinimidyl propionate)
EDC	(1-Ethyl-3-(3-dimethylaminopropyl) carbodiimide hydrochloride
EGFR	Epidermal growth factor receptor
ELISA	Enzyme-linked immunosorbent assay
EpCAM	Epithelial cell adhesion molecule
EFA1	Eukaryotic translation elongation factor 1 alpha 1
FB1	Fumonisin B1
FGR	Fetal growth restriction
FOLR1	Folate receptor alpha
g-CN	Carbon nitride
GFAP	Glial fibrillary acidic protein
GO	Graphene oxide
GPC1	Glypican-1
HER2	Human epidermal growth factor receptor 2
HITC	Hexamethyl-indotricarbocyanine iodide
HPV	Human papillomavirus
IgG	Immunoglobulin G
IL-6	Interleukin-6
IL-10	Interleukin-10
IR808	Indocyanine near-infrared dye derivative
LDHB	Lactate dehydrogenase B
LFA	Lateral flow assay
LNGFR	Low-affinity nerve growth factor receptor
LOD	Limit of detection
LPS	Lipopolysaccharide
LSPR	Localized surface plasmon resonance
ManLAM	Mannose-capped lipoarabinomannan
MBA	Mercaptobenzoic acid
MCAM	Melanoma cell adhesion molecule
MCSP	Melanoma-associated chondroitin sulfate proteoglycan
MGDTNB	Malachite green and DTNB combination
MGITC	Malachite green isothiocyanate
MI	Myocardial infarction
MIF	Macrophage migration inhibitory factor
MIP	Molecularly imprinted polymer
MMC	Malachite green carbinol
MMP-9	Matrix metalloproteinase-9
MMP-13	Matrix metalloproteinase-13
MMTAA	Mercaptomethylthioacetic acid
MOFs	Metal-organic frameworks
MPY	Mercaptopyridine
MUC16	Mucin-16
NBA	Nile Blue A
NB	Nile Blue
NBT	4-Nitrobenzenethiol
NHS	N-Hydroxysuccinimide
NIR	Near-infrared
NSE	Neuron-specific enolase
NS1	Non-structural protein 1
NSs	Nanostars
NPs	Nanoparticles
NR	Nanorods
NT-proBNP	N-terminal pro-B-type natriuretic peptide
OTA	Ochratoxin A
Ox-TMB	Oxidized tetramethylbenzidine
PB	Prussian Blue
PCA	Principal component analysis
PCTP	Pentachlorothiophenol
PD-L1	Programmed death-ligand 1
PEG	Polyethylene glycol
PI3K	Phosphoinositide 3-kinase
PLAP	Placental alkaline phosphatase
PLS-R	Partial least squares regression
ppb	Parts per billion
PP	Polypropylene
PPY	Polypyrrole
PS	Polystyrene
PSMA	Prostate-specific membrane antigen
Pt	Platinum
PVC	Polyvinyl chloride
R6G	Rhodamine 6G
RRMs	Raman reporter molecules
RS	Raman spectroscopy
Ru(bpy)_3_^2+^	Tris(2,2′-bipyridine)ruthenium(II)
SERS	Surface-enhanced Raman spectroscopy
SERS-LFA	SERS-based lateral flow assay
SERS-LFIA	SERS-based lateral flow immunoassay
SERS-PLFS	SERS paper lateral flow strip
SP10	Testis-specific acrosomal protein
SpIA	S-layer protein A
TBI	Traumatic brain injury
TEPSA	3-(Triethoxysilyl)propyl succinic anhydride
TFMBA	2,3,5,6-Tetrafluoro-4-mercaptobenzoic acid
THI	Thionine
TMV	Tobacco mosaic virus
TNF-α	Tumor necrosis factor-alpha
TSA	Thiosalicylic acid
TTC	Tetracycline
t-PSA	Total prostate-specific antigen
ToxB	Host selective toxin
UCH-L1	Ubiquitin carboxyl-terminal hydrolase L1
UTI	Urinary tract infection
VFA	Vertical flow assay
ZEN	Zearalenone
